# Surgical management and imaging evaluation of post-traumatic periscaphoid osteoarthritis (SLAC and SNAC wrists)

**DOI:** 10.1007/s00256-026-05128-z

**Published:** 2026-02-01

**Authors:** Hicham Bouredoucen

**Affiliations:** Division of Radiology, Department of Imaging and Medical Informatics, Geneva University Hospitals, University of Geneva, Rue Gabrielle-Perret-Gentil 4, 1211 Geneva 14 Geneva, Switzerland

**Keywords:** Scapholunate advanced collapse, Scaphoid nonunion advanced collapse, Pancarpal collapse, Wrist osteoarthritis, Scapholunate ligament injury, Scaphoid nonunion, Carpal imaging

## Abstract

An untreated scapholunate interosseous ligament injury or scaphoid nonunion leads to progressive post-traumatic periscaphoid osteoarthritis and carpal collapse. Symptomatic forms, known as scapholunate advanced collapse (SLAC) or scaphoid nonunion advanced collapse (SNAC), are common and disabling, requiring surgical management. Numerous operative options exist depending on the stage of collapse and the extent of joint involvement. We apply a modified Watson radiological classification consisting of four stages of pancarpal collapse, integrating the etiological origin (SLAC or SNAC) and the arthritic progression pattern. This review summarizes current surgical principles for each stage of the disease and highlights the key role of imaging in postoperative assessment. Radiography remains the first-line tool to evaluate carpal alignment and implant positioning. CT and CBCT provide high-resolution analysis of bony structures and fixation devices, while ultrasound enables dynamic evaluation of superficial soft tissues and implant–tendon relationships without metallic artifacts. MRI, despite its sensitivity for soft tissues and bone marrow, remains limited by metal-induced distortion. A comprehensive understanding of surgical techniques, biomechanical principles, and implant types is essential to accurately interpret postoperative outcomes and detect complications early.

## Introduction

**Fig. 1:**
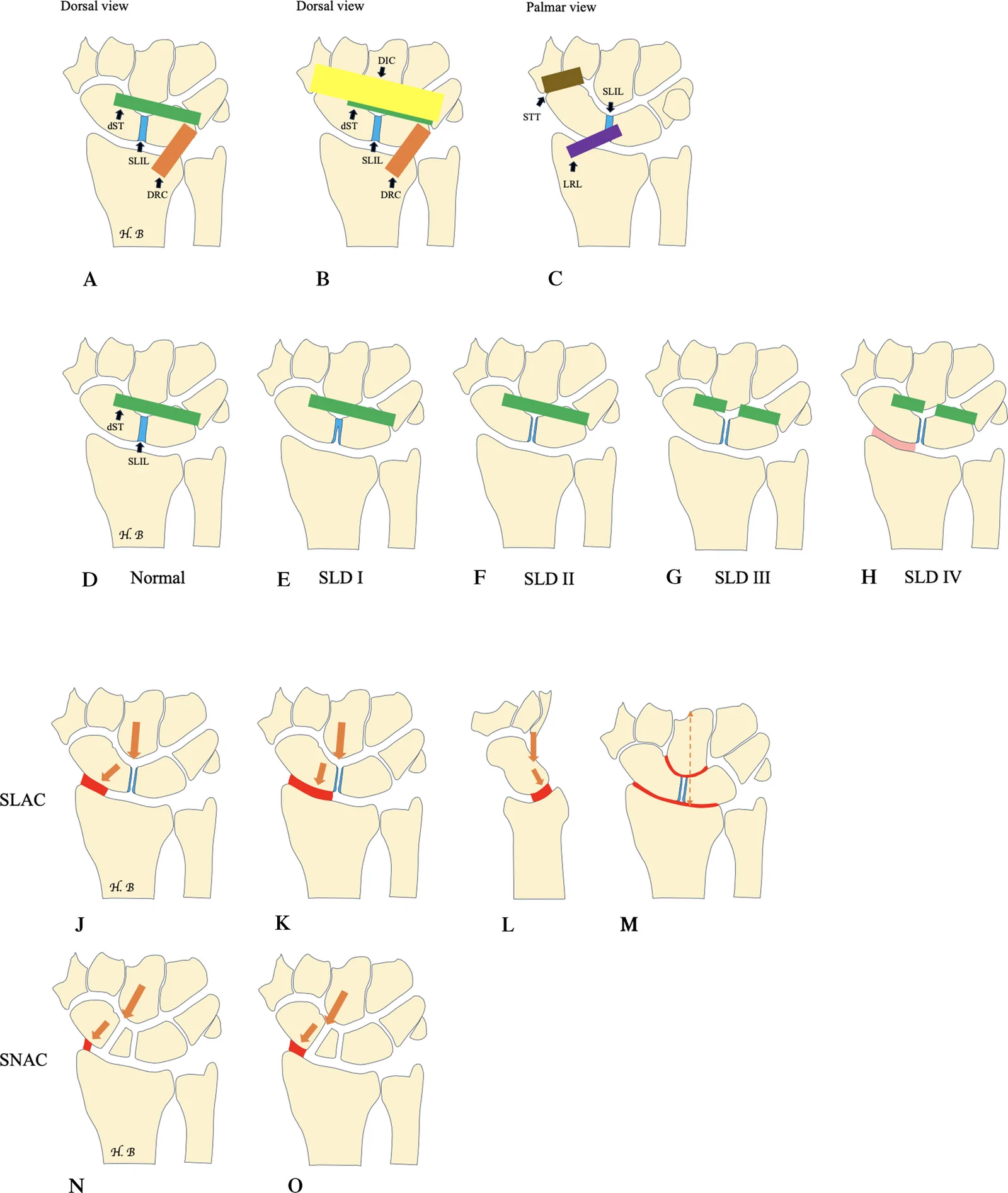
Schematic drawings illustrating scapholunate stability, stages of scapholunate dissociation, and biomechanical progression toward osteoarthritis in SLAC and SNAC wrists. (I) Anatomical and biomechanical stabilizers of the scapholunate complex (**A**–**C**). The stability of the scapholunate (SL) complex relies primarily on the scapholunate interosseous ligament (SLIL), particularly its dorsal component, which is biomechanically the most significant. Secondary stabilizers include dorsal ligaments—the dorsal intercarpal (DIC), dorsal scaphotriquetral (dST), and dorsal radiocarpal (DRC) ligaments—which limit dorsal translation and abnormal rotation of the scaphoid and lunate. Volar stabilizers include the scaphotrapeziotrapezoid (STT) ligament complex and the long radiolunate (LRL) ligament, which maintain volar alignment and regulate scaphoid and lunate rotation. Dynamic stabilization is provided by the wrist extensor muscles (extensor carpi radialis longus and brevis), flexor carpi ulnaris, and abductor pollicis longus, which actively contribute to carpal kinematics (tendons not depicted). (II) Stages of scapholunate dissociation and progression toward SLAC (**D**–**H**). For pedagogical purposes, only the dorsal scaphotriquetral (dST) ligament is depicted in panels **D**, **E**, **F**, **G**, and **H** as a representative secondary stabilizer of the scapholunate compartment. This schematic simplification does not imply an isolated or predominant biomechanical role of the dST ligament, but rather aims to facilitate visualization of the sequential failure of secondary stabilizing structures and its impact on progressive carpal malalignment. **D** Normal: intact SLIL and dST ligament, with normal carpal alignment and no scapholunate diastasis. **E** Stage I (pre-dynamic instability): Partial tear of the SLIL with an intact dST ligament. Carpal alignment remains normal, without scapholunate diastasis. **F** Stage II (dynamic instability): Complete rupture of the SLIL with preserved dST integrity. Transient scapholunate diastasis is observed only on stress radiographs, while static alignment is maintained. **G** Stage III (static instability): Complete SLIL rupture associated with partial or complete injury of the dST ligament. Persistent scapholunate diastasis (> 3 mm) and carpal malalignment are visible on standard radiographs. **H** Stage IV (advanced rotatory instability/SLAC wrist): Complete rupture of the dorsal and volar/intermediate components of the SLIL and the dST ligament. This results in scaphoid flexion, dorsal extension, and volar displacement of the lunate, capitolunate subluxation, a zigzag deformity known as dorsal intercalated segment instability (DISI), leading to progressive pancarpal osteoarthritis and loss of carpal height. (III) Biomechanics of carpal collapse and osteoarthritic progression in SLAC and SNAC wrists (**J**–**O**). In SLAC wrists (**J**–**M**), shear forces generated by the capitate act on the radial aspect of the proximal carpal row at the scapholunate junction (**J**), leading within months to styloid–radioscaphoid osteoarthritis (**K**) and degeneration between the proximal pole of the scaphoid and the posterior aspect of the radial scaphoid facet (**L**). In advanced stages, capitate migration into the scapholunate space results in pancarpal arthritis and collapse with loss of carpal height (**M**). In SNAC wrists (**N**–**O**), shear forces from the capitate act more laterally, across the scaphoid nonunion site (**N**), leading over 5–10 years to localized osteoarthritis between the radius and the distal scaphoid fragment (**O**)

Scapholunate (SL) instability is the most common form of dissociative carpal instability [1–4]. It is the primary cause of wrist osteoarthritis, referred to as scapholunate advanced collapse (SLAC) [3]. The stability of the SL complex [5] relies primarily on the scapholunate interosseous ligament (SLIL), particularly its dorsal portion, which is biomechanically the most significant [6–9]. Secondary stabilizers of the SL complex include, dorsally, the dorsal intercarpal (DIC), dorsal scaphotriquetral (dST), and dorsal radiocarpal (DRC) ligaments, which limit dorsal translation and rotation of the scaphoid and lunate; volarly, the scaphotrapeziotrapezoid (STT) and long radiolunate (LRL) ligaments, which maintain volar alignment and regulate scaphoid and lunate rotation; and dynamically, the wrist extensor muscles (extensor carpi radialis longus and brevis), the flexor carpi ulnaris, and the abductor pollicis longus, which actively contribute to the control of carpal kinematics (Fig. 1) [10–14].

Injury to the DIC ligament plays a pivotal role in the progression from dynamic to static SL instability [11–14].

**Fig. 2:**
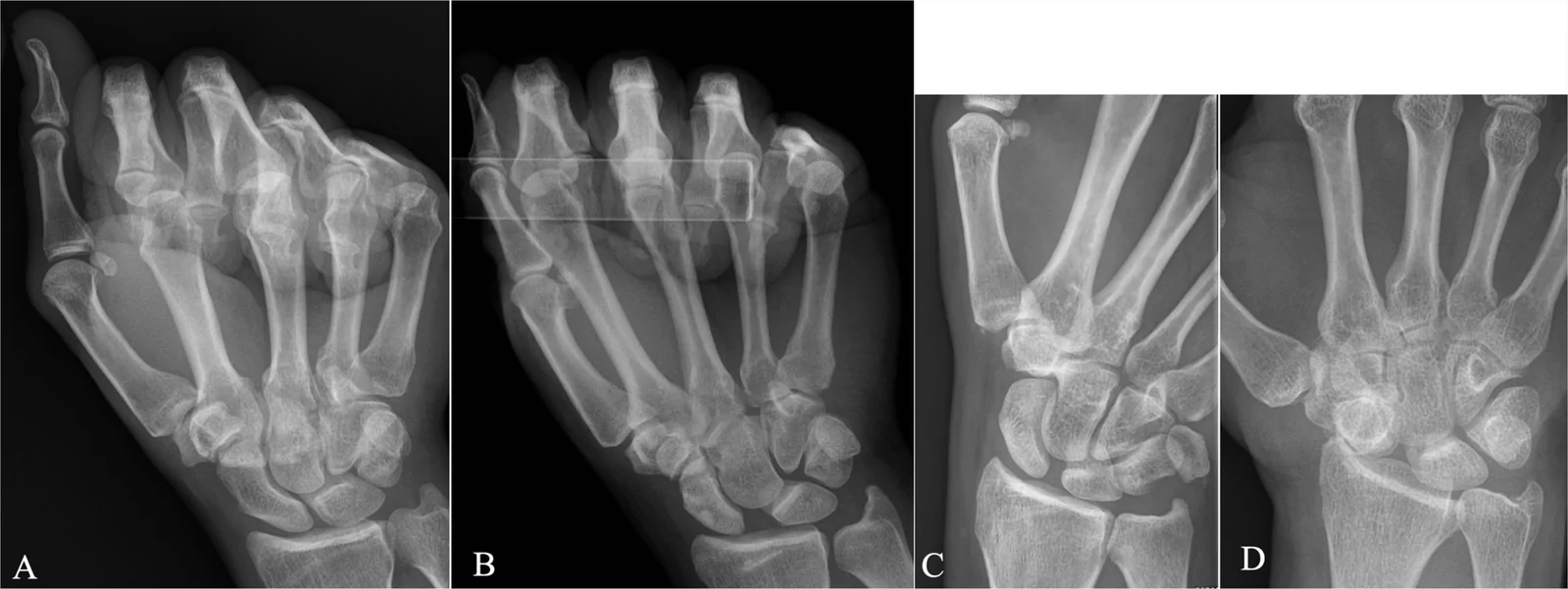
Radiographic spectrum of scapholunate instability. Posteroanterior (PA) radiographs were obtained in neutral (static) and stress positions. Static views assess carpal alignment and the scapholunate interval under physiological conditions, whereas stress views (pencil-grip view) apply functional loading to reveal dynamic instability. **A** Stage I—Pre-dynamic scapholunate instability: Stress PA radiograph shows no scapholunate diastasis (< 3 mm), with normal carpal alignment. Static radiographs appear normal. **B** Stage II—Dynamic scapholunate dissociation: Stress PA radiograph demonstrates a transient scapholunate diastasis (> 3 mm), indicating dynamic instability. Static views are often normal. **C** Stage III—Static scapholunate dissociation: Static PA radiograph shows persistent scapholunate diastasis (> 3 mm), consistent with established instability. **D** Stage IV—SLAC wrist: Static PA radiograph demonstrates degenerative changes within the radioscaphoid compartment, potentially progressing to carpal collapse.

SL instability represents a continuum ranging from occult partial tears to advanced carpal collapse (SLAC), resulting from combined failure of the SLIL and secondary stabilizing mechanisms [13, 14]. According to the modified Watson classification incorporating ligamentous assessment [13, 14], stage I (occult or pre-dynamic) is characterized by a partially injured or intact dorsal SLIL, an intact palmar/intermediate SLIL, and intact secondary stabilizers (DIC, dST, DRC, STT, LRL) (Fig. 1), with no SL diastasis (Fig. 2). Diagnosis at this stage relies on static radiographs, arthroscopy, or high-resolution magnetic resonance imaging (MRI) [15–17].

In stage II (dynamic instability), the dorsal SLIL may be partially or completely ruptured, the palmar/intermediate SLIL is often intact, and the secondary stabilizers show partial injury, particularly the DIC, while the dST, DRC, STT, and LRL may be partially injured or intact (Fig. 1). Diastasis is transient and only visible on stress radiographs (Fig. 2), which are complemented by arthroscopy, high-resolution MRI, or arthro-computed tomography when necessary [8, 13, 14, 17–19].

Stage III (static SL dissociation) is defined by complete rupture of the dorsal SLIL, with partial or complete involvement of the palmar/intermediate SLIL. Secondary stabilizers are significantly compromised, including a completely ruptured DIC, while dST and DRC may be partially injured, and STT and LRL are often intact or partially involved (Fig. 1). SL diastasis is present in static radiographs, with an SL gap exceeding 3 mm (Fig. 2) and an SL angle greater than 60°, and arthroscopy can be used for confirmation [2, 8, 13, 14, 17, 20–23].

Finally, stage IV corresponds to rotatory subluxation or dorsiflexed intercalated segment instability (DISI) advanced instability, where both dorsal and palmar/intermediate SLILs are completely ruptured, and all secondary stabilizers (DIC, dST, DRC, STT, LRL) are completely ruptured (Fig. 1). The detached lunate extends dorsally and shifts volarly, resulting in subluxation of the capitate over the posterior horn of the lunate [3]. This zigzag deformity is referred to as DISI [1]. SL diastasis is present along with scaphoid rotation (Fig. 2). Imaging includes static radiographs showing the ring sign and SL gap, dynamic radiographs to assess scaphoid rotation, computed tomography (CT), or arthroscopy if surgical planning is needed. This stage corresponds to SLAC, characterized by progressive carpal osteoarthritis starting at the radioscaphoid joint and eventually involving the entire carpus [13, 14]. Management depends on the stage of injury and the involved ligaments, ranging from arthroscopic repair of partial lesions to salvage procedures for advanced SLAC, including partial or total arthrodesis and arthroplasty according to alignment and residual function [14]. As long as SL instability is reducible (i.e., the scaphoid can be realigned), ligament reconstruction is indicated [23]. Various ligamentoplasty techniques are described, including free or pedicled tendon grafts and procedures using the extensor carpi radialis brevis, extensor carpi radialis longus, flexor carpi radialis, or scapholunate intercarpal ligamentoplasty (SLIC).

Scaphoid nonunion is defined as a lack of bony healing six months after fracture [24]. Causes include missed or delayed diagnosis, inadequate immobilization or surgical fixation, or inappropriate conservative management in displaced fractures. Avascular necrosis, especially in fractures of the proximal third, is also a key contributor [25]. The proximal pole is particularly vulnerable due to its limited retrograde blood supply from the distal pole [25, 26]. Nonunion typically follows a staged progression: initial resorption, bone demineralization, sclerosis of fracture margins, and possibly a “humpback deformity” [25]. Two types are described: stable, with fibrous bridging between fragments, and unstable, with interfragmentary fluid signal on MRI. In unstable nonunion, proximal scaphoid rotation and lunate extension generate a DISI pattern, leading to carpal collapse termed scaphoid nonunion advanced collapse (SNAC) [27]. Here, the capitate’s shearing force acts more laterally, across the fracture gap, and arthrosis remains limited for several years (typically 5–10) between the radius and distal scaphoid fragment (Fig. 1) [28]. Treatment is typically surgical, using techniques such as internal fixation alone or with vascularized or non-vascularized bone grafts [29–33].

In later stages, the radiographic features of SLAC and SNAC converge, although in SNAC, the proximal scaphoid often behaves similarly to the lunate (Fig. 1) [28]. In both patterns, radio–lunate joint involvement is rare and occurs only in advanced disease—its presence may preclude treatment options that rely on preserving this joint space.

**Fig. 3:**
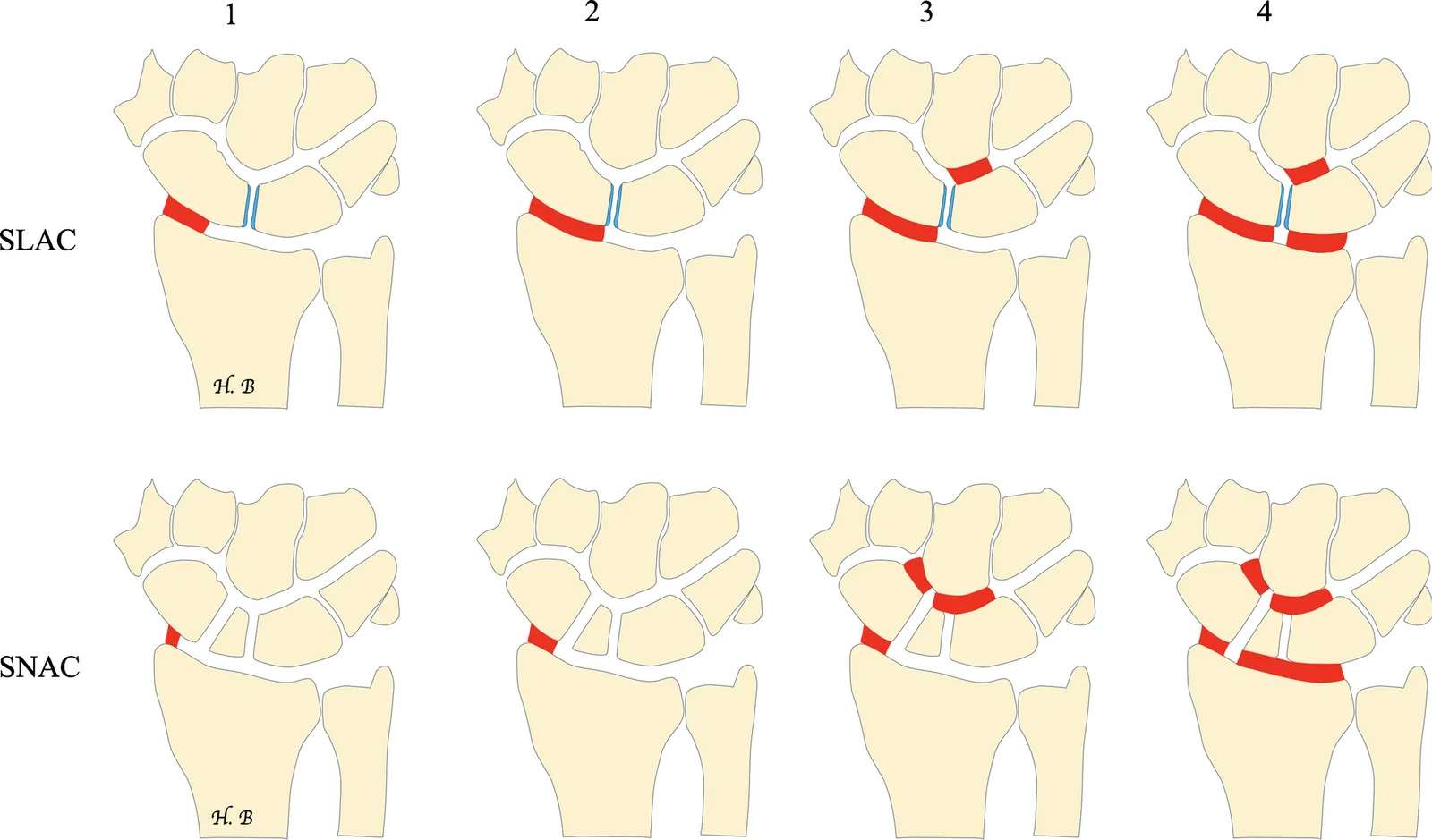
Stages of carpal collapse in SLAC and SNAC wrists, modified from Watson. Schematic drawings. SLAC, scapholunate advanced collapse; SNAC, scaphoid nonunion advanced collapse. In these schematics, osteoarthritic chondropathy is highlighted in red. SLAC: Carpal collapse secondary to rupture of the scapholunate interosseous ligament (SLIL). SNAC: Carpal collapse secondary to scaphoid nonunion. Stage 1: Chondropathy limited to the styloid–radioscaphoid junction. Stage 2: Chondropathy involving the entire radioscaphoid joint. Stage 3: Chondropathy affecting both the radioscaphoid and capitolunate joints. Stage 4: Advanced pancarpal chondropathy involving the radiolunate, radioscaphoid, and midcarpal joints

**Fig. 4:**
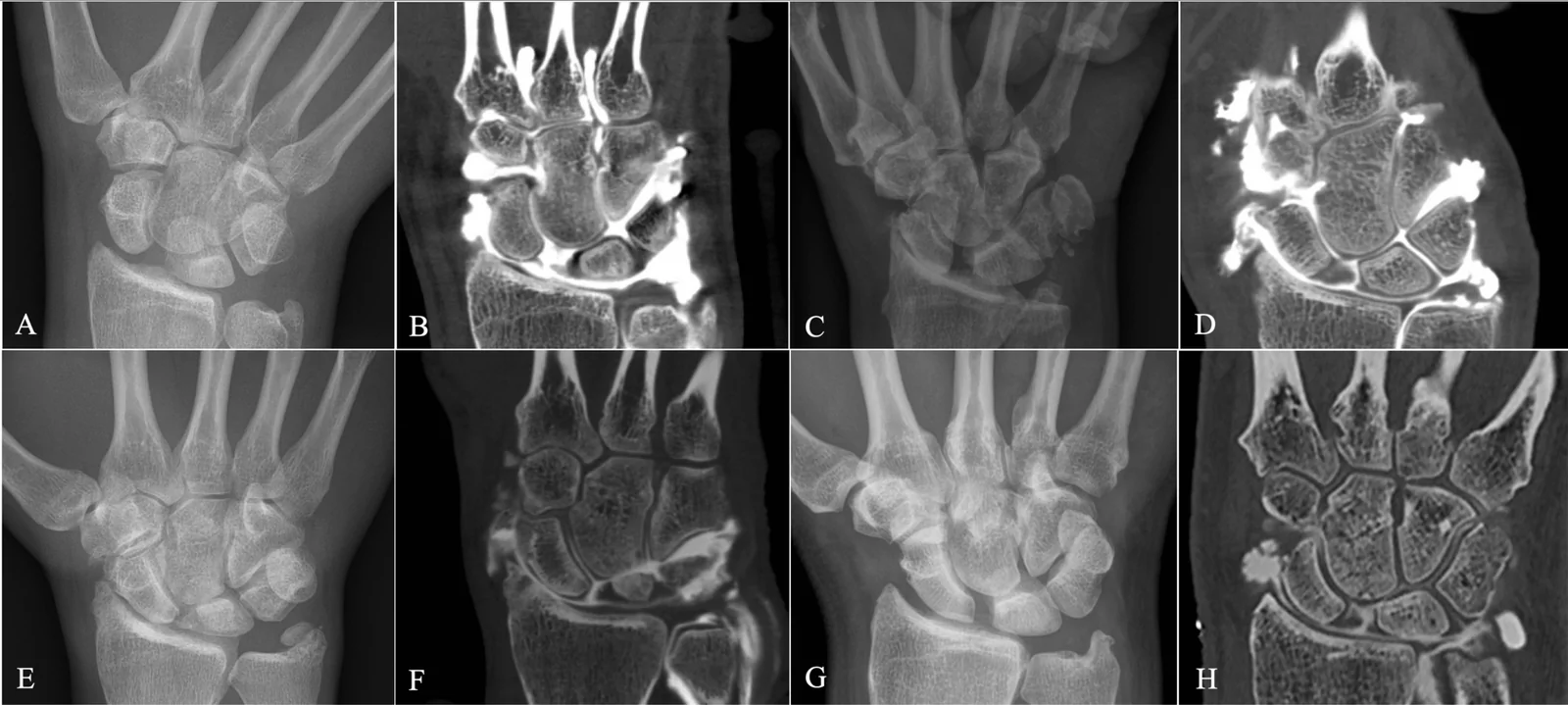
Radiographic and arthro-CBCT spectrum of scapholunate advanced collapse (SLAC) according to the Watson classification. This figure illustrates, using standard posteroanterior radiographs and arthro-CBCT, the progression of osteochondral lesions across the different SLAC stages, resulting from chronic scapholunate instability due to rupture of the scapholunate interosseous ligament. **A**, **B** SLAC stage 1: Standard posteroanterior radiograph (**A**) and arthro-CBCT (**B**) demonstrating early osteoarthritis limited to the styloid–radioscaphoid junction, with preservation of the midcarpal joint space. **C**, **D** SLAC stage 2: Osteoarthritic involvement extending to the entire radioscaphoid joint, clearly depicted on arthro-CBCT (**D**), while the capitolunate joint remains preserved. **E**,** F** SLAC stage 3: Combined radioscaphoid and capitolunate osteoarthritis, reflecting progression of carpal collapse, with loss of midcarpal congruence visible on both modalities. **G**, **H** SLAC stage 4: Advanced pancarpal osteoarthritis involving both the radiocarpal (radioscaphoid and radiolunate) and midcarpal compartments, corresponding to the terminal stage of collapse. This iconography highlights the complementary value of arthro-CBCT for detailed cartilage assessment and accurate staging of carpal osteoarthritis

**Fig. 5:**
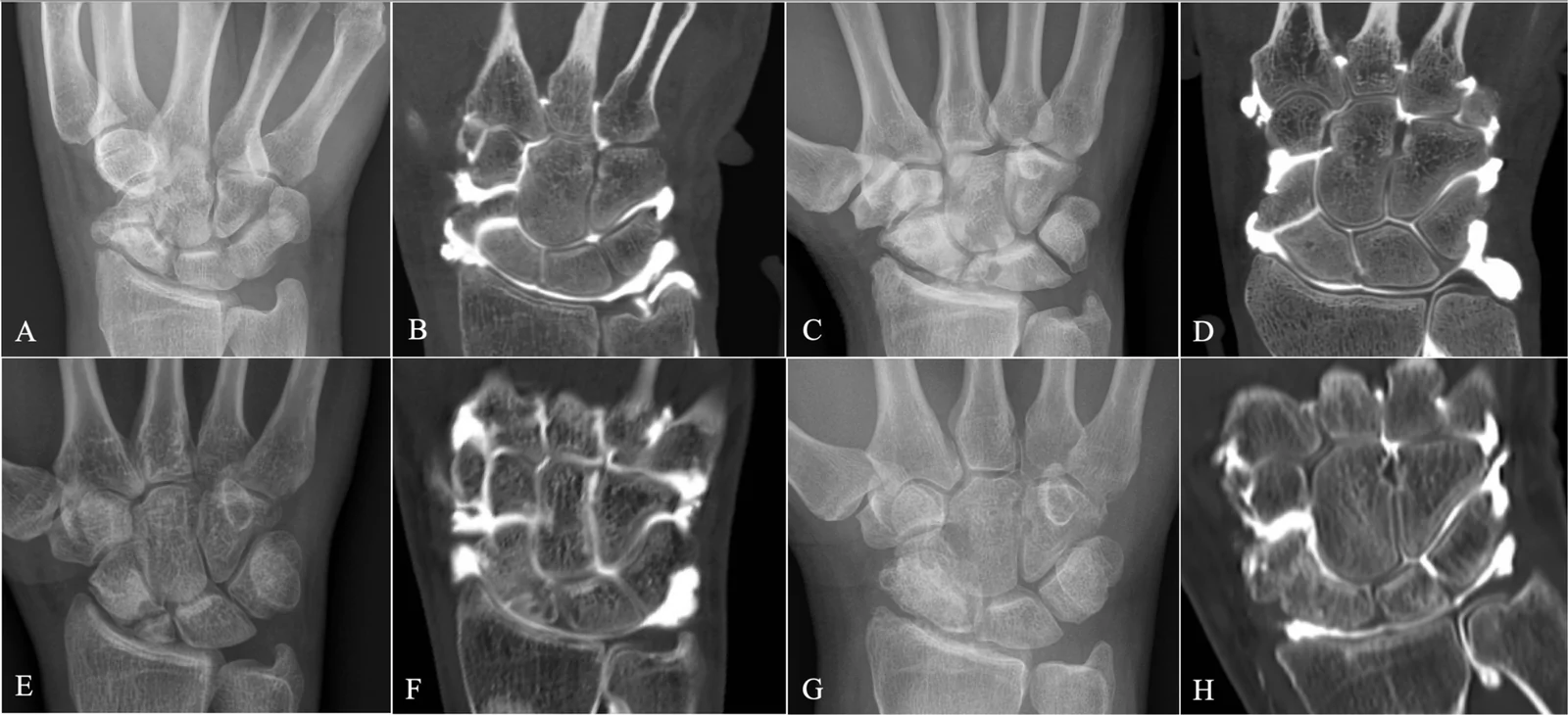
Radiographic and arthro-CBCT spectrum of scaphoid nonunion advanced collapse (SNAC) according to the Watson classification. This figure presents the different SNAC stages resulting from scaphoid nonunion, using standard posteroanterior radiographs and arthro-CBCT to illustrate the topographic progression of carpal osteoarthritis. **A**, **B** SNAC stage 1: Standard radiograph (**A**) and arthro-CBCT (**B**) demonstrating focal osteoarthritis limited to the styloid–radioscaphoid joint opposite the distal scaphoid fragment, with preservation of the remaining articular compartments. **C**, **D** SNAC stage 2: Extension of osteoarthritis to the entire radioscaphoid joint, while the capitolunate joint remains preserved. **E**, **F** SNAC stage 3: Combined involvement of the radioscaphoid and capitolunate compartments, reflecting progression of carpal collapse secondary to nonunion. **G**,** H** SNAC stage 4: Advanced pancarpal osteoarthritis involving both radiocarpal and midcarpal joints, corresponding to the terminal stage of the disease. Arthro-CBCT allows detailed evaluation of cartilage status and articular surfaces, facilitating stage differentiation and therapeutic decision-making

A schematic representation of the stages of carpal collapse in SLAC and SNAC wrists is shown in Fig. 3. Radiographic and arthro-CBCT illustrations detailing the progression of SLAC and SNAC stages are presented in Figs. 4 and 5.

This article outlines the surgical treatment principles adapted to each stage of post-traumatic periscaphoid osteoarthritis [28, 34], with a focus on post-therapeutic imaging modalities essential for postoperative monitoring and outcome evaluation.

Postoperative imaging assessment of periscaphoid osteoarthritis requires in-depth knowledge of surgical techniques and implanted materials to identify early complications. Conventional radiography remains the first-line modality for evaluating bone alignment and implant positioning, though it is limited in soft tissue analysis. High-resolution, especially dynamic, ultrasound enables detailed assessment of superficial musculoskeletal structures and implant–soft tissue interactions, unaffected by metal artifacts. CT and cone beam computed tomography (CBCT) provide excellent visualization of bone and surgical hardware, though metal artifacts may reduce diagnostic quality—this can be mitigated by optimized protocols. MRI offers detailed soft tissue and bone marrow assessment but is prone to distortion from metallic implants, despite artifact-reduction techniques. A tailored imaging strategy, aligned with the clinical scenario and surgical technique used, is essential for optimal patient management.

## Treatment and post-therapeutic imaging of SLAC 1/SNAC 1

**Fig. 6:**
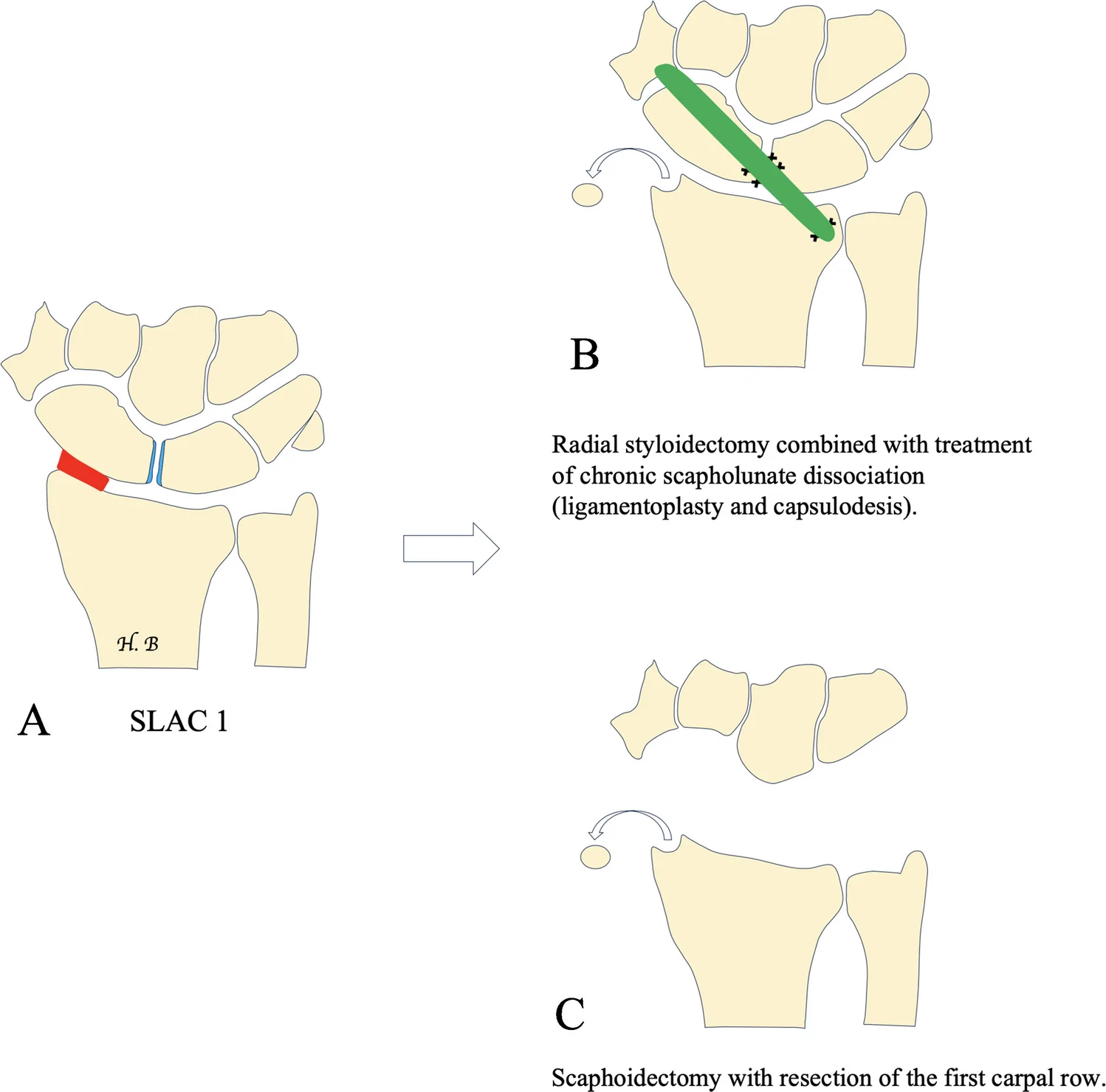
Therapeutic options for SLAC stage 1 wrists. Schematic illustration of SLAC stage 1, characterized by osteoarthritis limited to the styloid–radioscaphoid junction (**A**). Two surgical options are depicted. **B** Radial styloidectomy combined with treatment of chronic scapholunate dissociation by ligament reconstruction and capsulodesis, aiming to restore carpal stability and slow collapse progression. **C** Scaphoidectomy combined with proximal row carpectomy, representing a salvage procedure indicated according to cartilage involvement, patient age, and functional demands.

SLAC stage 1 refers to osteoarthritis localized between the radial styloid and the scaphoid (Fig. 3). This cartilage degeneration, situated between the proximal pole of the scaphoid and the posterior portion of the radial scaphoid facet, results from biomechanical alterations of the scaphoid, which disrupt the kinematics of the proximal carpal row. The standard treatment is conservative, typically consisting of a radial styloidectomy combined with management of chronic SL dissociation. A recent study demonstrated that arthroscopic radial styloidectomy yields favorable long-term functional outcomes, with sustained improvement in pain and wrist function, a low complication rate, and high patient satisfaction, suggesting that it may be considered prior to more invasive surgical interventions [35]. Due to ongoing uncertainties regarding optimal treatment of chronic SL dissociation, some surgical teams opt directly for scaphoidectomy with proximal row carpectomy (PRC) (Fig. 6), depending on the extent of arthrosis, the patient’s age, and the expected functional demand [28]. Evaluation of the arthritic stage of the scaphoid’s proximal pole is based on CT arthrography or MR arthrography (Fig. 3) [36].

**Fig. 7:**
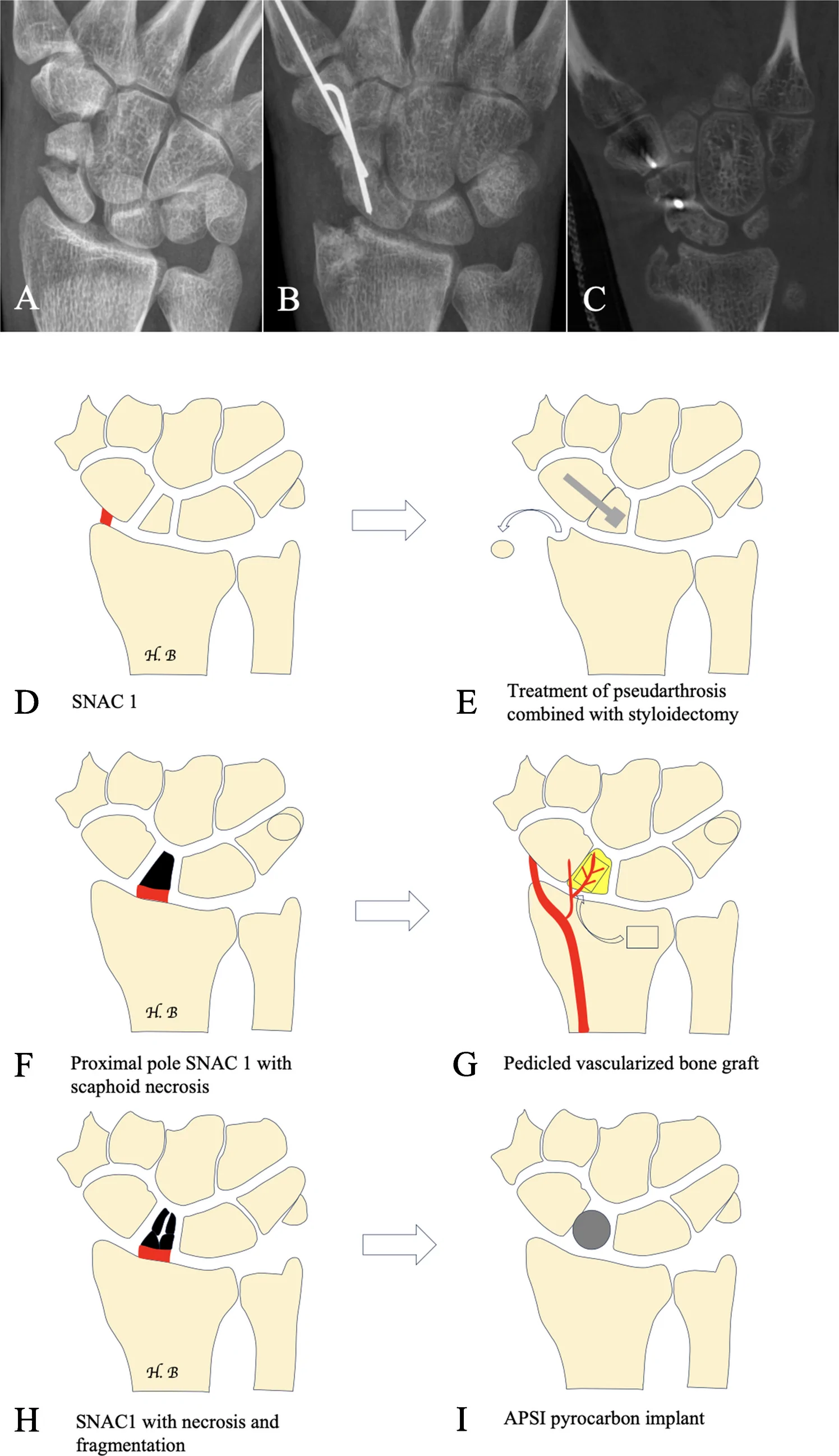
Imaging and therapeutic options for SNAC stage 1 wrists. Pre- and post-therapeutic imaging: **A** Standard posteroanterior radiograph showing scaphoid nonunion associated with SNAC stage 1, with osteoarthritis limited to the styloid–radioscaphoid joint. **B** Posteroanterior radiograph and **C** cone beam computed tomography (CBCT) following surgical treatment combining reconstruction of the nonunion with a vascularized bone graft harvested from the base of the ipsilateral second metacarpal, stabilized with Kirschner wires, and radial styloidectomy corresponding to resection of the arthritic radial zone. Schematic therapeutic options for SNAC stage 1:**D**–**I** Schematics illustrating different treatment strategies adapted to SNAC stage 1. **D**, **E** SNAC stage 1 with scaphoid nonunion treated by reconstruction combined with radial styloidectomy. **F**, **G** SNAC stage 1 involving the proximal scaphoid pole with osteonecrosis, treated by pedicled vascularized bone graft. **H**, **I** SNAC stage 1 with osteonecrosis and fragmentation of the proximal scaphoid pole, treated by implantation of a pyrocarbon APSI (adaptive proximal scaphoid implant)

SNAC stage 1 also involves osteoarthritis between the radial styloid and the scaphoid (Fig. 3), but in this case, the degenerative changes are directly related to the site of the nonunion. Cartilage damage generally remains focal for several years, limited to the distal radioscaphoid joint space, while the proximal articulation is preserved [28]. Treatment consists of addressing the scaphoid nonunion in combination with radial styloidectomy, which involves resecting the arthritic portion of the radial facet (Fig. 7) [37].

The study by Vutescu et al. [37] demonstrated that the location of scaphoid nonunion determines the indication for reconstruction combined with radial styloidectomy. Distal or waist nonunions are associated with limited cartilage wear (< 6 mm) and may be managed conservatively, whereas proximal pole nonunions result in more extensive cartilage degeneration (> 6 mm), exceeding the safe limit of resection and often requiring salvage procedures. These findings highlight the critical role of fracture location in surgical planning and in preserving wrist stability.

An arthroscopic approach allows minimization of procedural invasiveness while effectively relieving pain and providing sustained improvement in wrist function in patients with SNAC stage I [35].

Other therapeutic options may also be considered. In SNAC stage I cases involving the proximal pole of the scaphoid with osteonecrosis, a pedicled vascularized bone graft harvested from the dorsal aspect of the radius may be performed. In the presence of osteonecrosis with fragmentation, implantation of a pyrocarbon prosthesis such as the APSI (adaptive proximal scaphoid implant) represents an alternative treatment option (Fig. 7) [38, 39]. Isolated radial styloidectomy, without concomitant treatment of SL dissociation or scaphoid nonunion, may provide temporary pain relief; however, its indications remain limited, particularly when definitive treatment of the nonunion is not feasible [28, 40, 41]. Even in such cases, an arthroscopic approach reduces the risk of complications and secondary surgery, with reported follow-up demonstrating high patient satisfaction and a rapid return to activities of daily living [35].

Table 1 summarizes the therapeutic strategies and post-operative imaging modalities for SLAC/SNAC stage 1.

**Table 1:** SLAC/SNAC 1 [28, 35–41]

Stage	Primary treatment	Alternatives/options	Post-treatment imaging	Main complications
SLAC 1	Arthroscopic or open radial styloidectomy ± management of chronic scapholunate dissociation; scaphoid excision + proximal row carpectomy (PRC) for advanced osteoarthritis	PRC depending on age, functional demand, and extent of arthrosis	CT or MR arthrography to evaluate proximal scaphoid cartilage	Progression of osteoarthritis, persistent pain
SNAC 1	Radial styloidectomy combined with scaphoid nonunion reconstruction; arthroscopic approach preferred when feasible	Pedicled vascularized bone graft for proximal pole osteonecrosis; APSI (pyrocarbon implant) for fragmentation; conservative management for distal/waist nonunions with limited cartilage wear (< 6 mm)	CT or MR arthrography for distal radioscaphoid joint evaluation	Non-union-related complications, residual pain, risk of salvage procedures in proximal pole nonunion
Notes	Isolated radial styloidectomy may provide temporary relief; arthroscopic approach reduces invasiveness and complication risk, improves functional outcomes and patient satisfaction			

## Treatment and post-therapeutic imaging of SLAC 2/SNAC 2

**Fig. 8:**
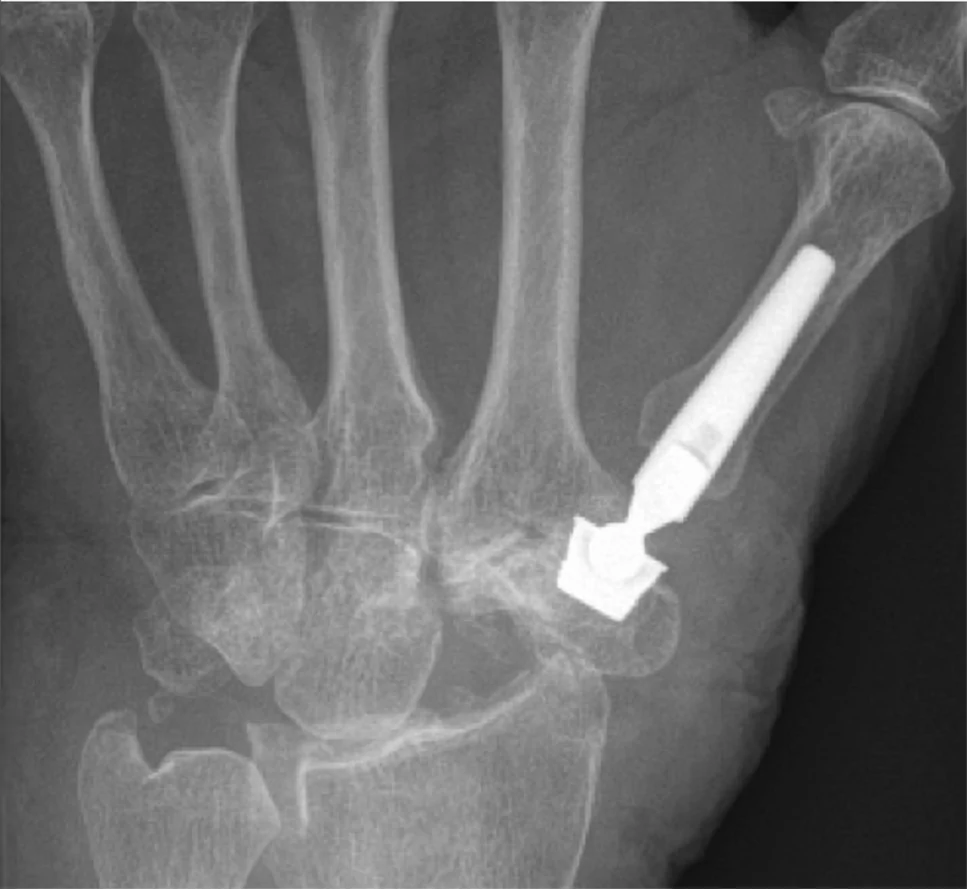
Resection of the proximal carpal row for a SNAC stage 2 wrist. Standard posteroanterior radiograph demonstrating a total trapeziometacarpal joint prosthesis

SLAC/SNAC stage 2 corresponds to osteoarthritis involving the entire radioscaphoid joint (Fig. 3). The primary treatment is resection of the proximal carpal row (Fig. 8). However, this procedure is contraindicated in the presence of capitolunate arthritis [42].

**Fig. 9:**
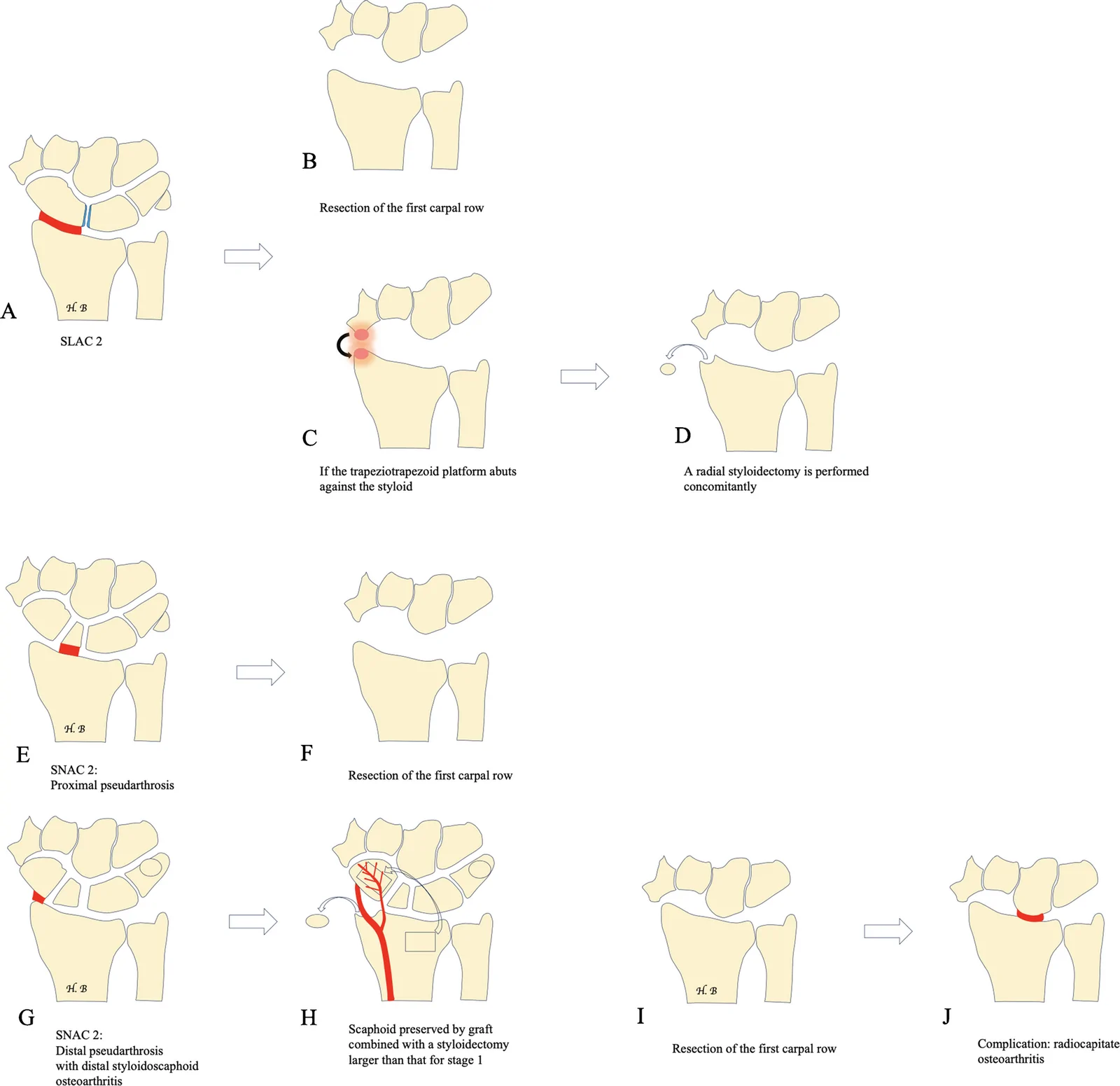
Therapeutic options for SLAC and SNAC stage 2 wrists and complications related to proximal row carpectomy. **A**–**D** SLAC stage 2 wrist.**A** Schematic illustration of SLAC stage 2, characterized by osteoarthritis involving the entire radioscaphoid joint with initial preservation of the capitolunate joint. **B** Reference treatment by proximal row carpectomy. **C**, **D** When the trapeziotrapezoid platform conflicts with the radial styloid during radial deviation and wrist extension, a posterolateral radial styloidectomy is performed to prevent residual impingement while preserving the volar radioscaphocapitate ligament. **E**–**H** SNAC stage 2 wrist. **E**, **F** Schematic of SNAC stage 2 related to proximal scaphoid nonunion, treated by proximal row carpectomy as the standard approach. **G**,** H** SNAC stage 2 secondary to distal scaphoid nonunion associated with styloid–radioscaphoid osteoarthritis. In this situation, the scaphoid may be preserved through bone graft reconstruction combined with a more extensive radial styloidectomy than that performed at stage 1. **I**,** J** Postoperative complication. **I** Schematic after proximal row carpectomy. **J** Illustration of radiocapitate osteoarthritis, the most frequent complication following proximal row carpectomy, resulting from incongruence between the distal radial articular surface and the capitate head

In cases of SLAC 2, PRC is considered the reference treatment. Recent data from a large multicenter cohort confirm that PRC results in an early and clinically meaningful improvement in patient-reported pain and function, with benefits maintained for at least 12 months postoperatively [43]. A posterolateral radial styloidectomy may be added when the trapeziotrapezoid platform impinges against the radial styloid during radial deviation and wrist extension (Fig. 9). During the procedure, care is taken to preserve the palmar radioscaphocapitate ligament [44]. In addition, denervation of the dorsal capsule through resection of the posterior interosseous nerve is routinely performed to prevent neuroma formation at closure [28].

From a functional standpoint, PRC allows a rapid reduction in pain, exceeding the minimal clinically important difference within the first 3 postoperative months, with continued improvement up to 12 months, while preserving most wrist ranges of motion and allowing grip strength to return to preoperative levels [43]. A biomechanical cadaveric model analysis demonstrated that PRC results in a significant increase in wrist contact forces, which may explain the development of progressive radiocapitate osteoarthritis [45]. This relatively predictable recovery trajectory makes PRC particularly well suited for shared decision-making and expectation management in active patients with SLAC 2 [43].

In SNAC 2 (Fig. 9), when the nonunion is proximal, PRC remains the standard approach. Functional outcomes after PRC in SNAC 2 appear comparable to those observed in SLAC 2, with similar improvements in pain and functional scores [43]. However, in cases of distal nonunion associated with stylo-scaphoid arthritis, the scaphoid may be preserved through bone grafting, combined with a more extensive radial styloidectomy than that performed in stage 1 [28].

Progressive radiocapitate osteoarthritis is the most frequent complication following PRC (Fig. 9), resulting from incongruity between the distal radial surface and the capitate [45–48]. Despite this potential radiographic progression, the overall complication rate after PRC remains moderate (approximately 10–11%), and conversion to salvage surgery (wrist arthroplasty or arthrodesis) is uncommon during the first postoperative year [43]. In addition to persistent pain, other potential complications include postoperative soft tissue infection, hematoma, or tendon injury [47].

**Fig. 10:**
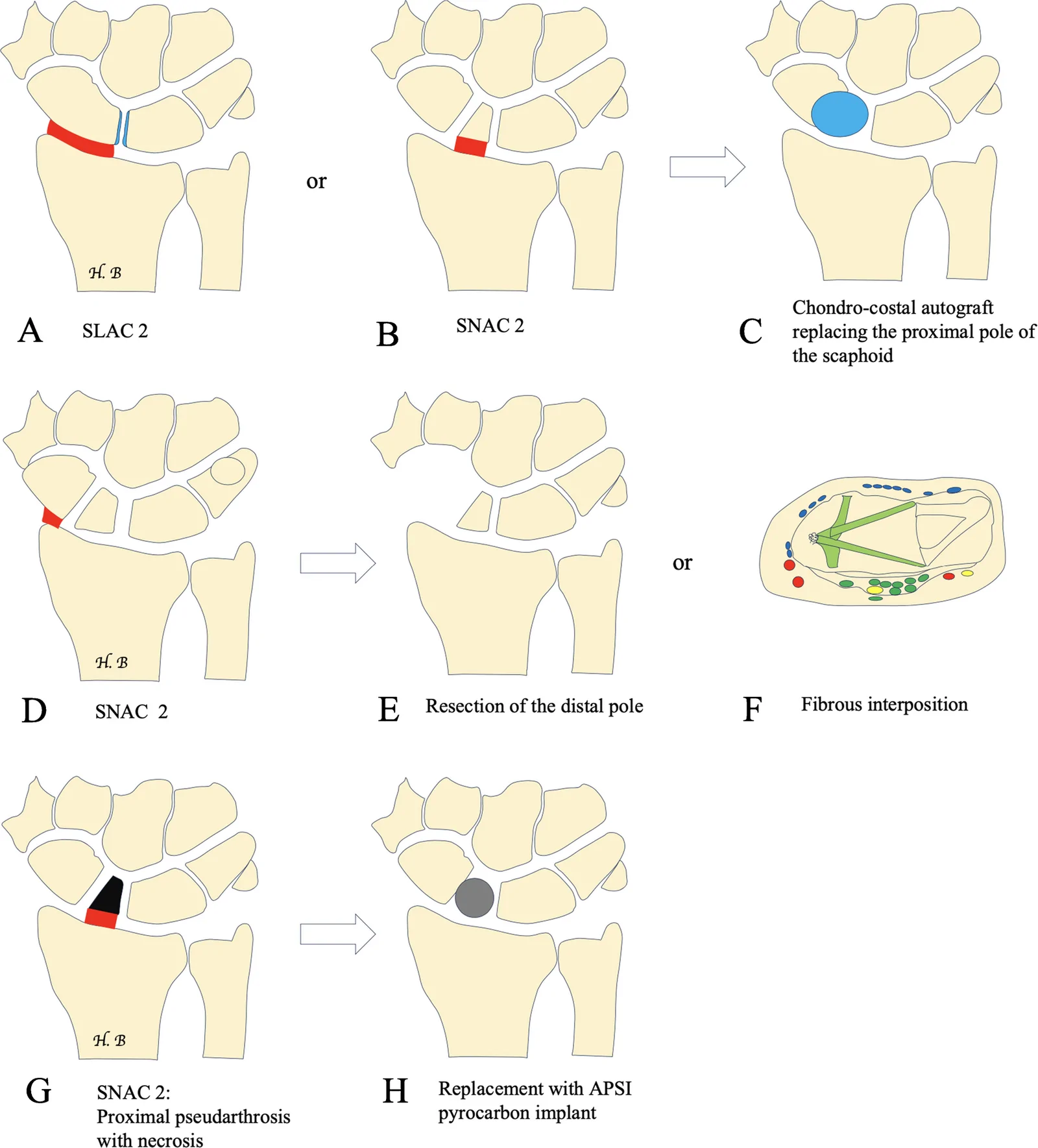
Alternative surgical options to the reference treatment in SLAC and SNAC stage 2 wrists. **A**–**C** SLAC 2 and SNAC 2 wrists. **A** Schematic of a SLAC stage 2 wrist and **B** of a SNAC stage 2 wrist. **C** In selected cases, replacement of the proximal scaphoid pole with an autologous costochondral graft to restore an articular surface and preserve carpal height. **D**–**F** SNAC stage 2 with distal scaphoid involvement. **D** Schematic of a SNAC stage 2 wrist. **E** Treatment by distal scaphoid pole resection. **F** Alternative treatment using fibrous interposition aimed at eliminating arthritic conflict between the distal scaphoid and the capitate. These techniques carry a risk of increased carpal collapse due to loss of scaphoid height. **G**,** H** SNAC stage 2 with proximal nonunion complicated by osteonecrosis. **G** Schematic illustrating proximal scaphoid nonunion with necrosis. **H** Treatment by replacement of the proximal pole using a pyrocarbon APSI implant, restoring carpal height and limiting collapse progression

**Fig. 11:**
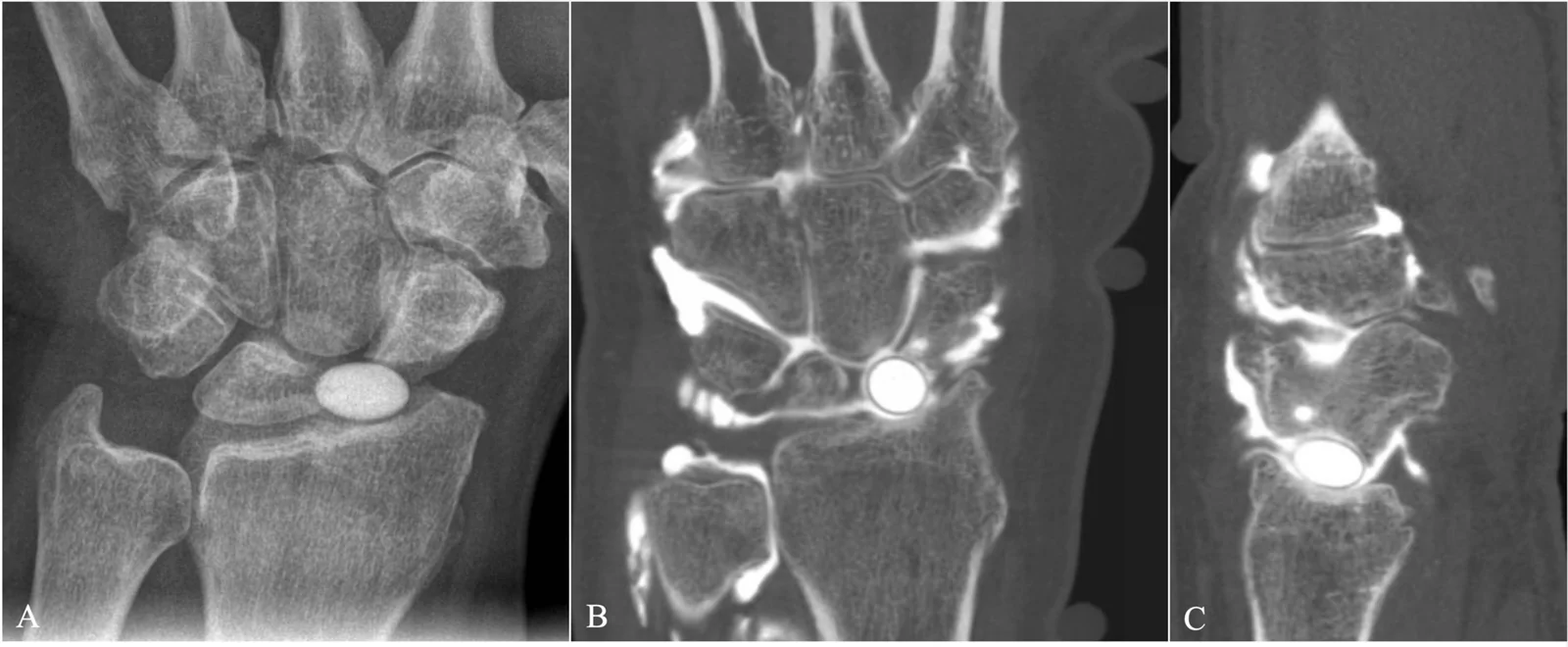
Replacement of the proximal scaphoid pole with an APSI (pyrocarbon implant) for a SNAC stage 2 wrist with proximal nonunion and osteonecrosis. **A** Standard posteroanterior radiograph. **B** Coronal arthro-CBCT reconstruction. **C** Sagittal arthro-CBCT reconstruction performed 1 year after implantation, demonstrating failure of the APSI implant with development of radioscaphoid and midcarpal osteoarthritis involving both the prosthetic surfaces and the lunocapitate articulation

Among alternative treatment options (Fig. 10), chondrocostal autograft implantation into the proximal pole of the scaphoid has been proposed for SLAC 2 and SNAC 2 [49–51]. Some authors also recommend, in SNAC 2, resection of the distal pole of the scaphoid [52, 53] or fibrous tissue interposition [54], aiming to eliminate arthritic conflict between the distal scaphoid and the capitate. However, the main limitation of these techniques is the risk of worsening carpal collapse due to loss of scaphoid height, which normally contributes to stabilization of the midcarpal joint [28]. By contrast, PRC is supported by more robust evidence based on validated functional scores and standardized assessments at multiple postoperative follow-up intervals [43]. Finally, in cases of SNAC 2 with proximal pole nonunion and associated osteonecrosis, implantation of a pyrocarbon APSI (Adaptive Proximal Scaphoid Implant) may be considered (Fig. 11) [55, 56].

Table 2 provides an overview of treatment options and radiological follow-up for SLAC/SNAC stage 2.

**Table 2:** SLAC / SNAC 2 [28, 43–56]

Stage	Primary treatment	Alternatives/options	Post-treatment imaging	Main complications
SLAC 2	Proximal row carpectomy (PRC) ± posterolateral radial styloidectomy; preservation of the radioscaphocapitate ligament	Dorsal denervation, costal cartilage autograft	Standard radiographs; CT for complications or follow-up	Progressive radiocapitate osteoarthritis, infection, hematoma, tendon injury
SNAC 2	PRC for proximal nonunion; distal nonunion with stylo-scaphoid arthritis: bone graft + extended styloidectomy	Distal scaphoid resection, fibrous tissue interposition, APSI (pyrocarbon implant)	Standard radiographs; CT	Risk of carpal collapse, progressive osteoarthritis, persistent pain
Notes	PRC provides rapid and clinically meaningful functional improvement, recovery of grip strength, moderate complication rate (10–11%), rare conversion to salvage surgery	Alternative procedures may increase risk of carpal collapse; individual choice depends on nonunion location and osteonecrosis		

## Treatment and post-therapeutic imaging of SLAC 3/SNAC 3

SLAC/SNAC stage 3 corresponds to osteoarthritis involving both the entire radioscaphoid joint and the capitolunate joint (Fig. 3). From stage 3 onwards, the anatomoradiological characteristics of SLAC and SNAC converge, and so do their treatments.

**Fig. 12:**
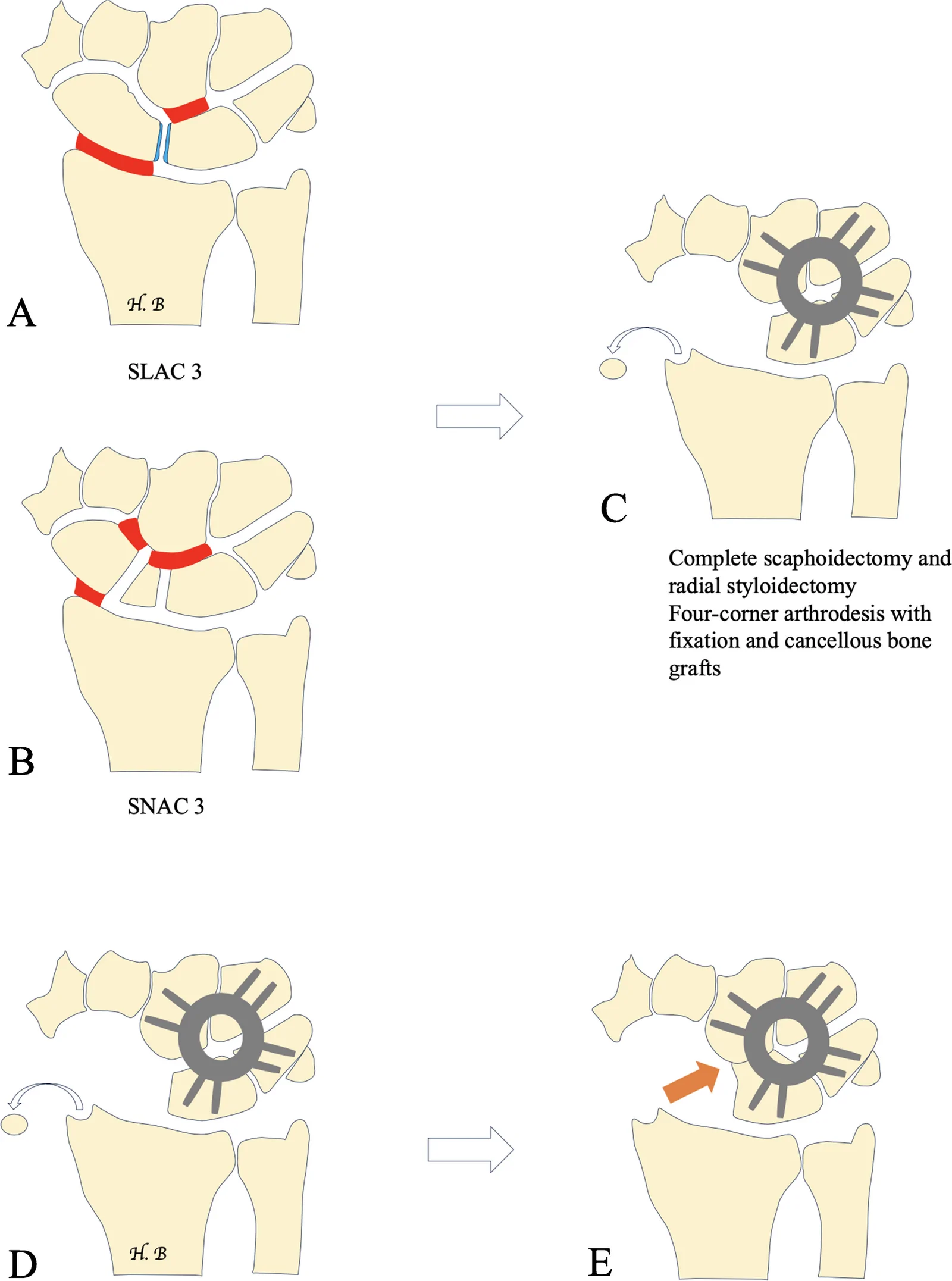
Treatment options for SLAC stage 3 and SNAC stage 3 wrists. **A**,** B** Schematics of SLAC stage 3 and SNAC stage 3 wrists. **C** Schematics illustrating complete scaphoidectomy and radial styloidectomy as preparatory steps for partial carpal arthrodesis. Four-corner arthrodesis with fixation and cancellous bone graft placement between debrided joint surfaces stabilizes the medial carpus and corrects DISI deformity. **D**,** E** Assessment of fusion following complete scaphoidectomy with four-corner arthrodesis. Successful fusion is defined by bridging trabeculae across the capitolunate interface (arrow E) and absence of pain or motion at the fusion site

**Fig. 13:**
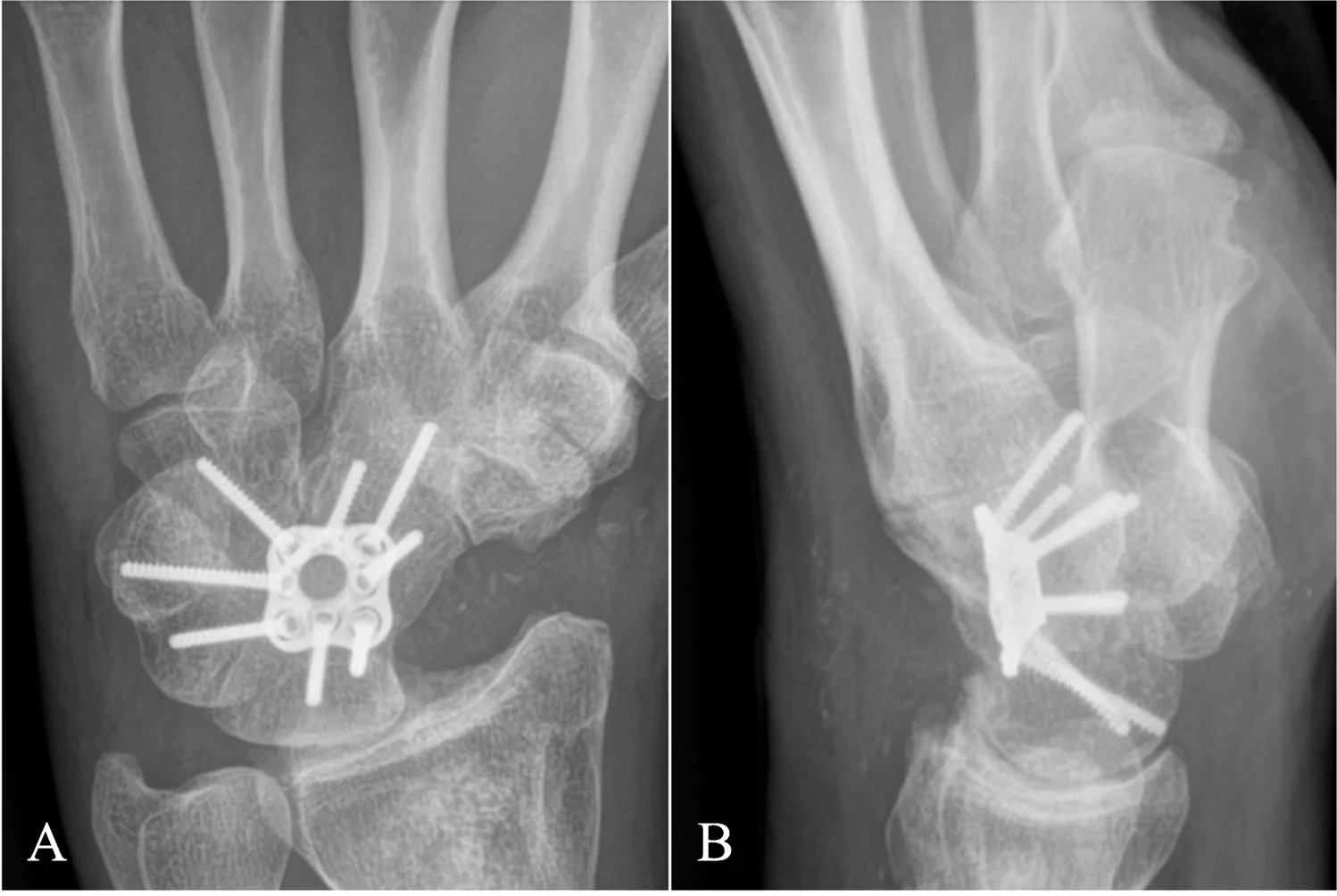
Radiological evaluation after scaphoidectomy with four-corner arthrodesis for a SLAC stage 3 wrist. **A**,** B** Standard posteroanterior and lateral radiographs. The dorsal plate is centered over the four fused carpal bones (triquetrum, lunate, capitate, hamate), ensuring appropriate midcarpal alignment. Neutral midcarpal alignment is essential to prevent dorsal radiocarpal impingement. The plate is embedded just beneath the dorsal carpal cortex to avoid conflict with dorsal soft tissues

Involvement of the capitolunate joint contraindicates PRC. However, recent evidence indicates that PRC may provide favorable functional outcomes and a lower complication profile compared with four-corner arthrodesis in selected patients [57]. Van Hernen et al. reported that PRC is associated with lower reoperation rates and a reduced risk of conversion to total wrist arthrodesis, particularly when *anterior interosseous nerve/posterior interosseous nerve* neurectomy is performed, while smoking increases the risk of secondary arthrodesis [58]. Treatment consists of complete scaphoid resection associated with debridement of the lunocapitate, hamatocapitate, and triquetrohamate joints, as well as DISI correction. Stabilization of the medial carpus is achieved through partial arthrodesis, for which many variations exist. The most classic and oldest technique is scaphoidectomy combined with four-corner fusion, i.e., arthrodesis of the four remaining carpal bones: triquetrum, capitate, lunate, and hamate (Fig. 12) [59, 60]. After scaphoidectomy, a radial styloidectomy is performed. Cancellous bone grafts, harvested from the resected scaphoid, distal radius, or iliac crest, are placed between the debrided joint surfaces [28]. Traditional fixation techniques (Kirschner wires, headless screws, standard staples) ensure effective consolidation with nonunion rates of 3–18%, but often require secondary removal and carry risks of infection, migration, and tendon irritation [60, 61]. Recent studies show that non-locking plates (spider plate) have higher rates of dorsal impingement, loosening, and nonunion (25–63%), whereas locking plates (flower plate) provide reliable consolidation with fewer postoperative complications and comparable functional outcomes [62]. Acar et al. [63] described a modern arthroscopic four-corner arthrodesis technique using two headless screws through an expanded midcarpal radial portal, allowing complete scaphoid fragment removal and DISI correction, with excellent functional results. Goyal et al. [64] proposed a cerclage technique for four-corner arthrodesis providing circumferential compression, avoiding dorsal cartilage surfaces and improving sagittal alignment, with a reduction in dorsal conflicts. Radiological evaluation of scaphoidectomy with four-corner fusion is performed with standard posteroanterior and lateral wrist radiographs and, to a lesser extent, CT. On normal postoperative radiographs, the dorsal plates are centered over the four fused carpal bones. The midcarpal joint must exhibit neutral alignment to avoid dorsal radiocarpal impingement [47, 65]; the plate is embedded just beneath the dorsal carpal cortex to avoid dorsal conflict (Fig. 13) [60, 66]. Ideally, dorsal plates should be fixed with two screws in each of the four fused carpal bones (particularly the lunate and capitate) [60].

**Fig. 14:**
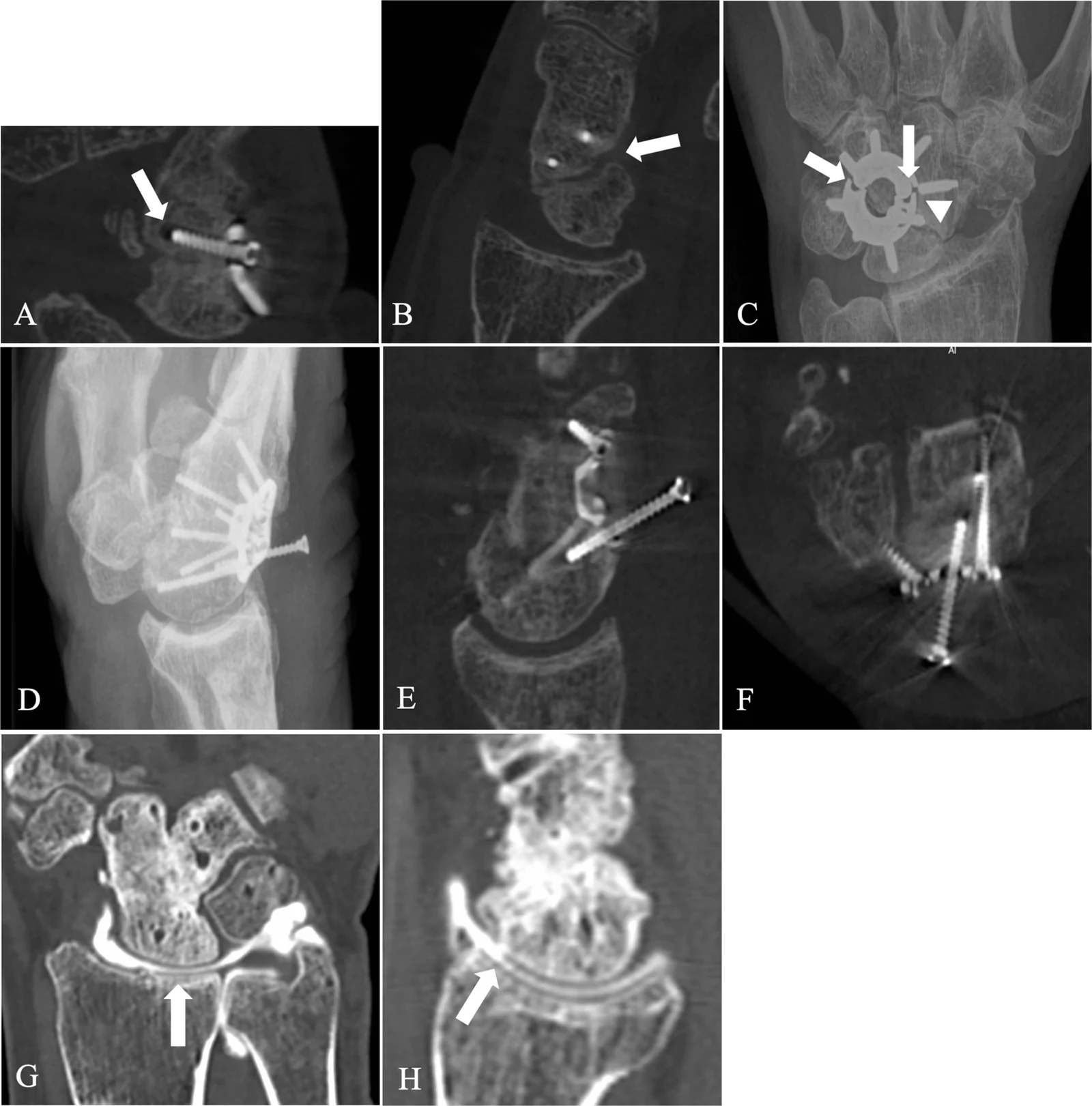
Potential complications following four-corner arthrodesis. **A**,** B** Sagittal CBCT reconstructions showing loosening and posterior backing out of the proximal capitate screw (arrow **A**), leading to partial lunocapitate fusion and widening of the capitolunate joint space (arrow **B**). **C** Posteroanterior radiograph demonstrating dorsal plate fracture (arrows) and capitate screw avulsion (arrowhead). **D**–**F** Standard lateral radiograph (**D**) and sagittal and axial CBCT reconstructions (**E**,** F**) showing posterior migration of a lunocapitate screw causing dorsal impingement. **G**,** H** Coronal and sagittal arthro-CBCT reconstructions demonstrating the development of dorsal radiocarpal osteoarthritis (arrows). These images provide a pedagogical overview of mechanical and degenerative complications relevant for radiologists and surgical planning

Following scaphoidectomy with four-corner fusion, the wrist is immobilized in a cast for 4 to 8 weeks to allow fusion of the capitolunate joint. Capitolunate fusion alone yields positive clinical outcomes, regardless of the fusion status of the hamate or triquetrum [60, 67]. Recent evidence suggests that limited arthrodeses, such as two-corner arthrodesis or three-corner arthrodesis, can provide pain relief and functional preservation comparable to four-corner arthrodesis, with similar complication rates, especially when modern compression screws are used [57, 68]. A thorough clinical examination and radiographs, sometimes complemented by CT, are used to assess healing. Although no universally accepted criteria exist to define fusion, it can be confirmed by the appearance of trabecular bridging at the capitolunate interface (Fig. 12) combined with the absence of localized pain or motion at the fusion site [60, 69]. CT is the most reliable imaging modality to assess bony consolidation, especially in the presence of metallic implants. Trabecular bridging across the midcarpal fusion site typically forms within 6 to 16 weeks [69]. One study reported a longer mean time to consolidation (6 months) using the Medartis Aptus Four-Corner Fusion plate. Reported fusion rates with traditional techniques, the Spider Limited wrist arthrodesis system, and the Xpode dorsal locking circular plate are 82–97%, 37–100%, and 80–96%, respectively [60, 67, 70]. Hayes et al. [71] demonstrated that overall radiographic union rates for all fixation methods reach 97%, with minimal clinical differences in range of motion and grip strength. Complications after four-corner arthrodesis remain significant and include nonunion, partial fusion, delayed union, pseudarthrosis, implant-related issues, carpal misalignment, radiolunate arthritis, radial styloid impingement, infections, soft tissue complications, and donor site morbidity (Fig. 14) [62–64, 68, 72].

**Fig. 15:**
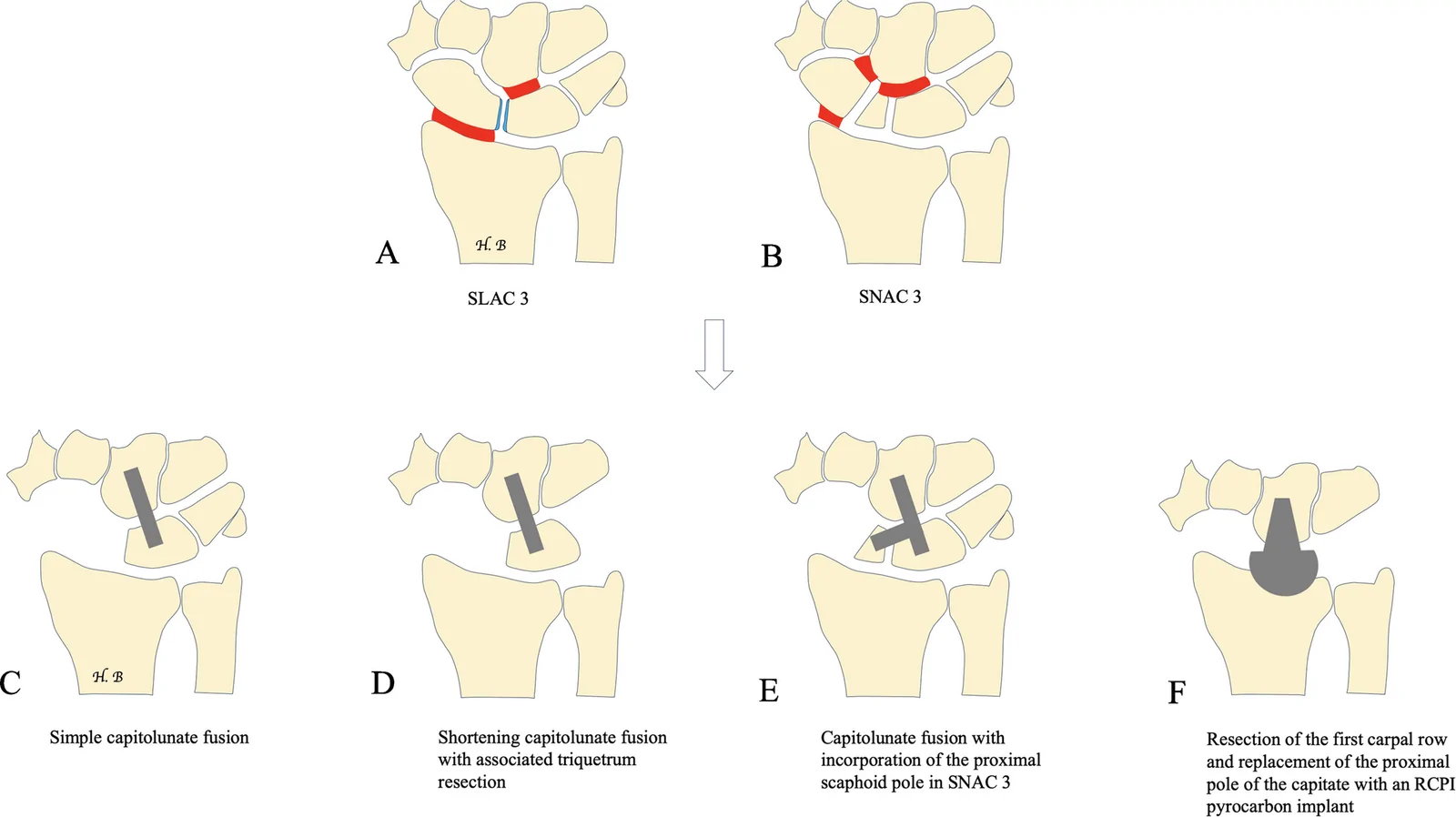
Surgical options for SLAC/SNAC stage 3 wrists. **A**,** B** Schematics of SLAC stage 3 and SNAC stage 3 wrists. **C** Simple capitolunate fusion, indicated when the lunate has adequate bone quality and joint surface contact. **D** Shortened capitolunate fusion with triquetrum resection, reducing pain and preserving motion at the expense of weakening the ulnar anti-translation ligament sling. **E** Capitolunate fusion incorporating the proximal scaphoid pole, specific to SNAC stage 3, restoring medial carpal stability. **F** Proximal row carpectomy with replacement of the proximal capitate pole using a pyrocarbon RCPI implant, illustrating technique selection based on wrist morphology and osteoarthritis stage

**Fig. 16:**
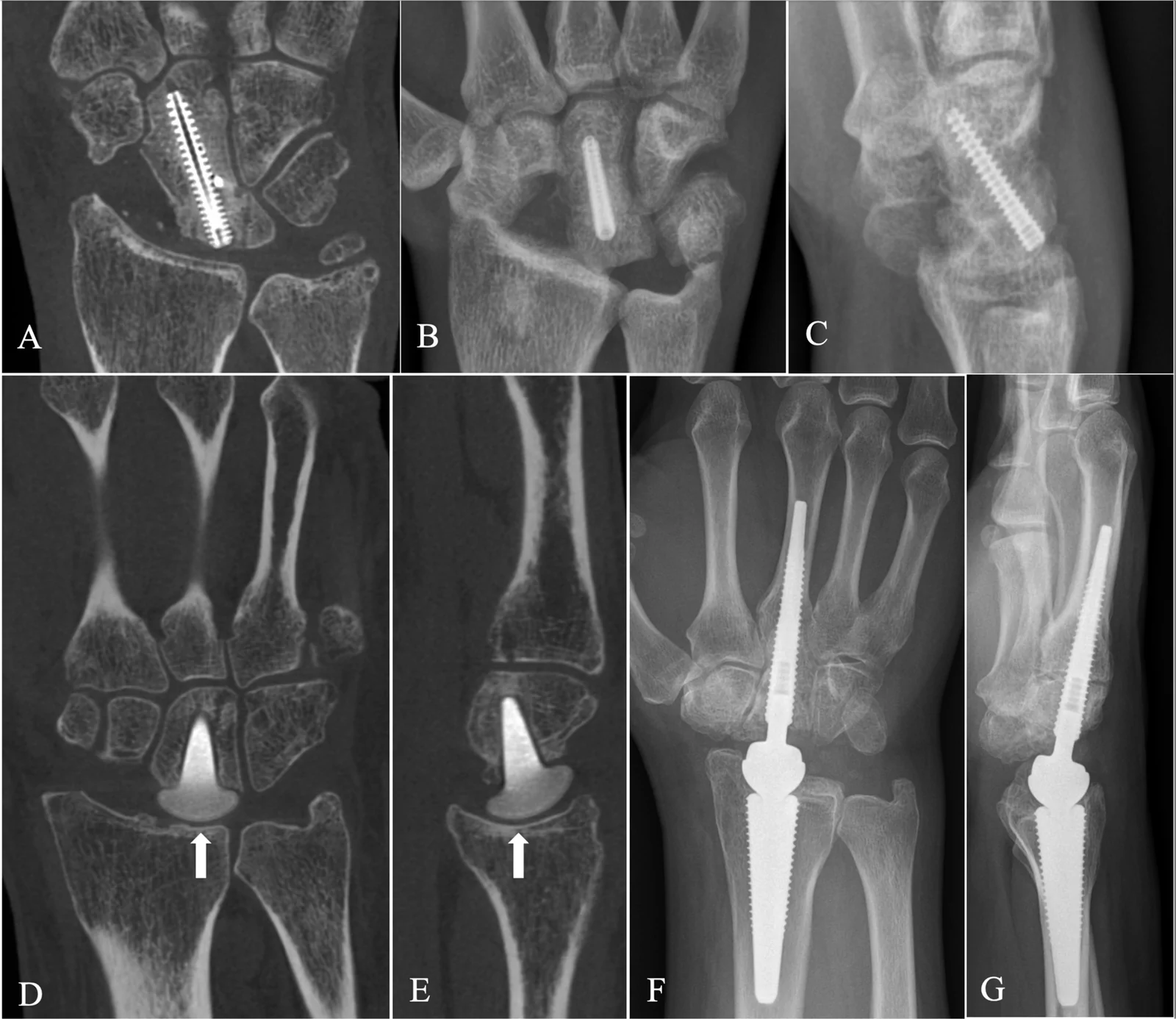
Clinical and radiological examples of treatment for SLAC/SNAC stage 3 wrists.** A**–**C** SLAC stage 3 wrist treated by scaphoidectomy and simple capitolunate fusion. Coronal CBCT reconstruction (**A**) and standard posteroanterior (**B**) and lateral (**C**) radiographs demonstrate central dorsal plate positioning and early capitolunate fusion. **D**,** E** SNAC stage 3 wrist treated by scaphoidectomy and simple capitolunate fusion. Coronal (**D**) and sagittal (**E)** CBCT reconstructions confirm fusion and DISI correction. **F**,** G** SNAC stage 3 wrist treated by proximal row carpectomy with a pyrocarbon RCPI implant. Complication showing chondropathy of the radial surface and prosthesis–radius arthropathy (arrows), subsequently managed with total wrist arthroplasty

Other treatment options (Fig. 15) relate to the specific joints to be arthrodesed [28]. Simple capitolunate fusion (two-corner arthrodesis) is effective if the lunate has good bone quality and contact surfaces (Fig. 16**)**, while three-corner arthrodesis may be particularly beneficial in type II lunates to avoid ulnotriquetral conflict [57, 68]. A simple capitolunate fusion is possible if the lunate has good bone quality and sufficient contact surfaces. A shortened capitolunate fusion with resection of the triquetrum is also possible [66, 67], but this resection weakens Kuhlmann’s ulnar anti-translation ligament sling. Capitolunate fusion incorporating the proximal pole of the scaphoid can be performed in SNAC 3. PRC can also be extended by replacing the proximal pole of the capitate with a pyrocarbon RCPI (resurfacing capitate pyrocarbon implant) [73, 74]. However, because the artificial bearing surface is small, this implant may not always be well tolerated during strenuous activity (Fig. 16).

PRC continues to offer advantages in terms of lower reintervention rates and reduced conversion to total wrist arthrodesis, especially when combined with selective *anterior interosseous nerve/posterior interosseous nerve* neurectomy and careful preservation of the dorsal capsule [57, 58].

In this context, a recent nationwide American analysis demonstrated a significant shift in surgical practice for the management of wrist osteoarthritis. Between 2009 and 2019, the utilization of PRC increased substantially, whereas four-corner fusion remained largely stable. Conversely, total wrist arthroplasty declined markedly, despite the introduction of fourth-generation implants, and rates of total wrist arthrodesis remained unchanged [75]. Although this study did not specifically differentiate post-traumatic etiologies, these trends reflect a growing preference for simpler, reliable procedures that partially preserve wrist mobility, directly relevant to the management of intermediate-stage SLAC and SNAC wrists.

In conclusion, current evidence-based recommendations suggest the following:

- PRC: functional benefit, fewer complications, rare conversion to arthrodesis [57, 58].
- Four-corner arthrodesis: classical reference technique, reliable consolidation, mild mobility restriction, locking plate fixation recommended to reduce complications [62, 64].
- Two-corner arthrodesis/three-corner arthrodesis: valid alternatives, comparable to four-corner arthrodesis functionally, preserving range of motion and reducing the risk of ulnotriquetral conflict [57, 68].

Table 3 details surgical techniques and post-treatment imaging assessment for SLAC/SNAC stage 3.

**Table 3:** SLAC/SNAC 3 [28, 57–75]

Stage	Primary Treatment	Alternatives/Options	Post-Treatment Imaging	Main Complications
SLAC/SNAC 3	Scaphoid excision + four-corner fusion (triquetrum, capitate, lunate, hamate) ± radial styloidectomy; autologous bone graft; fixation with K-wires, headless screws, or plates	PRC (proximal row carpectomy) with proximal capitate replacement (RCPI); limited arthrodeses: 2-corner (capitolunate) or 3-corner; modern plates (Spider®, Flower®, Aptus®, Xpode®); arthroscopic four-corner fusion with cerclage screw	PA and lateral radiographs; CT for trabecular consolidation and midcarpal alignment	Non-union, partial fusion, delayed consolidation, pseudarthrosis, DISI deformity, radiolunate osteoarthritis, implant-related complications, dorsal conflict, radial styloid impingement, infections, soft tissue complications, donor site morbidity
Notes	Immobilization 4–8 weeks; typical consolidation 6–16 weeks; PRC: functional benefit, fewer complications, rare conversion to arthrodesis; limited arthrodeses: preserve range of motion and reduce ulnotriquetral conflict			

## Treatment and post-therapeutic imaging of SLAC 4/SNAC 4

SLAC/SNAC stage 4 corresponds to advanced osteoarthritis involving both the radiocarpal joint (involving the radioscaphoid and radiolunate joints) and the midcarpal joint; the distal radioulnar joint may also be affected (Fig. 3) [28, 76, 77].

**Fig. 17:**
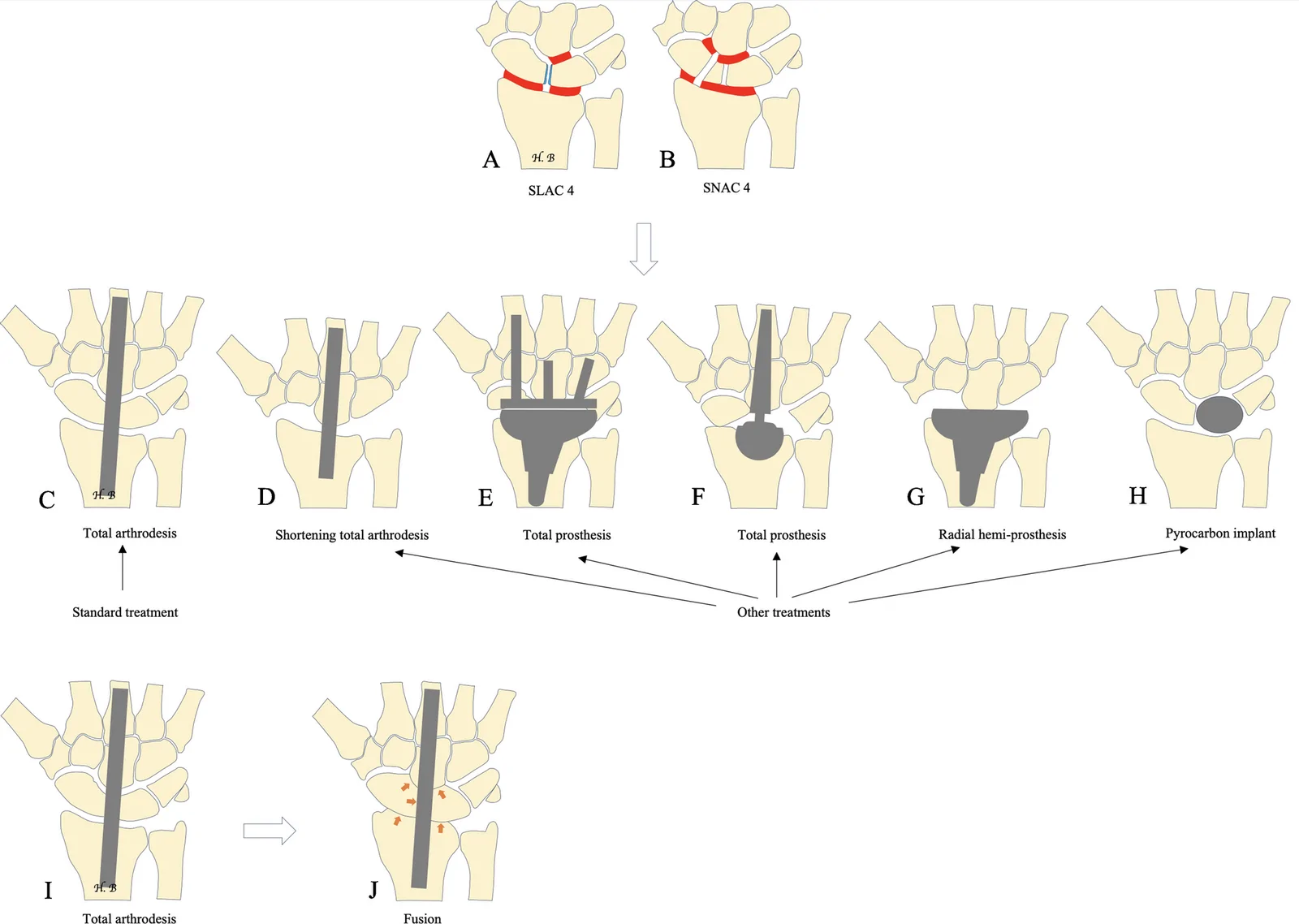
Therapeutic options for SLAC/SNAC stage 4 wrists. Schematic illustrations present the different treatment options for advanced periscaphoid osteoarthritis affecting the radiocarpal and midcarpal joints. **A**,** B** Schematics of SLAC stage 4 and SNAC stage 4 wrists. **C** Standard treatment: total wrist arthrodesis, providing rigid fusion of radiocarpal and midcarpal joints with loss of motion but durable pain relief and improved functional strength. **D**–**H** Alternative treatment options: **D** shortening total wrist arthrodesis, typically performed after failed proximal row carpectomy. **E**,** F** Total wrist arthroplasty aiming to preserve motion while reducing pain. **G** Radial hemiarthroplasty articulating with the capitate to limit carpal component loosening. **H** Massive pyrocarbon implant offering a compromise between pain relief and partial motion preservation. **I**,** J** Radiographic criteria of osseous fusion following total wrist arthrodesis, demonstrating homogeneous bony continuity and joint stability

Total wrist arthrodesis remains the reference standard treatment at this stage of osteoarthritis, particularly in patients with high functional demands or extensive pan-carpal destruction (Fig. 17). Older techniques, such as distal longitudinal corticocancellous bone graft “sliding” or fixation with pins, have largely been replaced by rigid fixation using a preformed dorsal plate positioned in slight wrist extension of approximately 10° [28, 76, 77]. A thin precontoured plate is typically secured with screws to the radius, capitate, and third metacarpal [28]. Adjunctive bone grafting is commonly performed and may be harvested from the iliac crest or obtained during concomitant PRC, improving fusion rates and providing autologous structural support [78, 79]. The joints most frequently included in the fusion mass are the radioscaphoid, radiolunate, SL, scaphocapitate, and lunocapitate joints [80]. Fusion of the carpometacarpal joint remains debated and should be tailored to clinical symptoms and preoperative imaging findings rather than performed routinely [78, 81]. Consolidation rates are high (96 to 100%), with radiographic union generally achieved within 6 months [47, 56, 82]. A systematic review by Ruskin et al. [78] comparing wrist fusions with and without concomitant PRC demonstrated similarly high union rates (97.7% vs. 96.2%, *P* = 0.17), with no statistically significant difference between the two techniques. Subgroup analysis of post-traumatic arthritis showed a non-significant trend toward higher union rates with PRC (98.3% vs. 94.4%). Total wrist arthrodesis provides reliable pain relief and improves grip strength [56]; however, comparative functional analyses consistently demonstrate that, despite excellent pain control, the complete loss of wrist motion negatively impacts activities requiring precision, adaptability, and positioning of the hand in space, even with preserved forearm rotation and compensatory elbow and shoulder motion [28, 74, 83]. Compared with arthrodesis in other joints, this loss of motion is generally considered less disabling due to these compensatory mechanisms [82]. Patient-reported outcome measures generally favor motion-preserving procedures when revision surgery is not required, particularly for tasks involving fine motor control and functional reach. Patient satisfaction rates range from 80 to 100% [56].

**Fig. 18:**
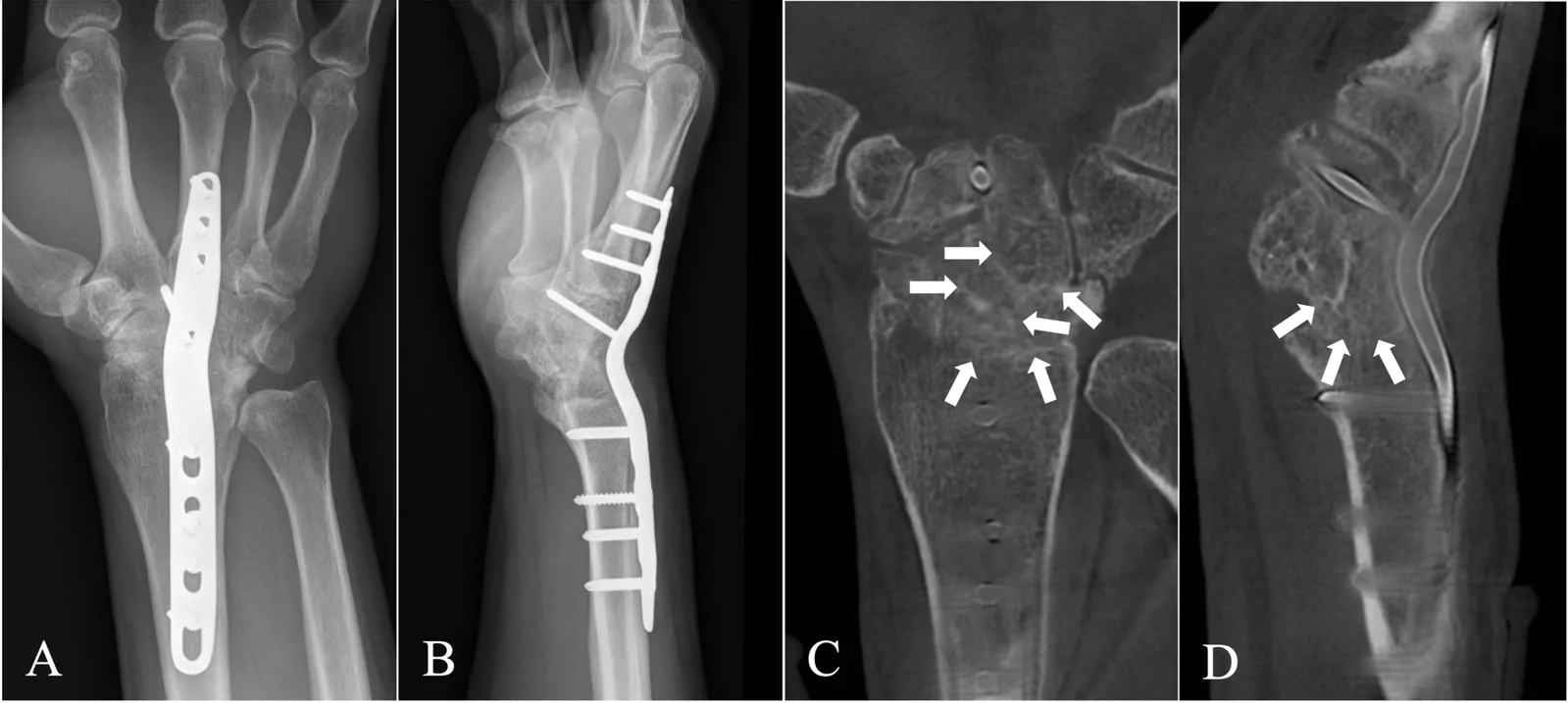
Total wrist arthrodesis: standard radiographic and postoperative CBCT imaging. **A**,** B** Standard posteroanterior (**A**) and lateral (**B**) radiographs showing fixation with a preformed dorsal plate, wrist positioned in slight extension (~ 10°), correct plate alignment, and absence of osseous displacement. **C**,** D** Coronal (**C**) and sagittal (**D)** CBCT reconstructions confirming solid fusion of the radioscaphoid, radiolunate, scapholunate, scaphocapitate, and lunocapitate joints (arrows). No abnormal mobility or periprosthetic radiolucent line is observed, consistent with satisfactory consolidation. The dorsal plate is flush with the cortical bone, minimizing soft-tissue irritation

Postoperative imaging typically demonstrates solid fusion of the radioscaphoid, radiolunate, SL, scaphocapitate, and lunocapitate joints, while fusion of the carpometacarpal joint remains inconsistently performed. Total wrist fusion should ideally be achieved with approximately 10° of dorsiflexion to optimize grip strength, particularly in non-rheumatoid patients [77]. The dorsal plate should remain flush with the dorsal cortical bone to reduce soft-tissue irritation (Fig. 18) [47]. Dorsal plating provides a rigid mechanical environment for healing, allowing compression across the fusion site and shorter postoperative immobilization, although plating can be associated with hardware-related complications such as screw fractures or plate loosening [78].

Complications of total wrist arthrodesis include pseudarthrosis [76]; sensory nerve complications such as neuroma formation or hypoesthesia of dorsal branches of the radial or ulnar nerves (2 to 5%) [84]; carpal tunnel syndrome (up to 10% of cases, with approximately half requiring surgical release) [84]; infections [76]; and hardware-related complications such as migration, loosening, or fracture [76]. Ulnocarpal impaction may occur, particularly when fusion is performed with excessive ulnar deviation, and can be treated with ulnar shortening osteotomy or distal ulnar excision [85]. Additional complications include distal radioulnar joint osteoarthritis [84], hematoma, major skin dehiscence (20–30%), infection, extensor tendon adhesions [47, 85], and symptomatic dorsal plates’ prominence causing painful irritation, bursitis, or extensor tenosynovitis, reported in up to two-thirds of patients and frequently requiring hardware removal [85]. Complex regional pain syndrome occurs in approximately 2–3% of cases [84].

Locked intramedullary wrist arthrodesis has recently emerged as an alternative to dorsal plating, aiming to reduce soft tissue irritation and hardware prominence. Walker et al. [86] reported on nine patients with post-traumatic and inflammatory arthritis treated with this system. Radiocarpal fusion was achieved in eight of nine patients after the initial procedure, with one patient achieving union after revision with bone grafting. The most common complication was distal screw migration, affecting three patients, leading to three revisions. Additional complications included metacarpal fracture with radiocarpal nonunion and symptomatic carpometacarpal nonunion. Despite acceptable fusion rates, the system demonstrated high complication and revision rates, highlighting limitations in distal fixation and the potential challenge of implant removal after union.

**Fig. 19:**
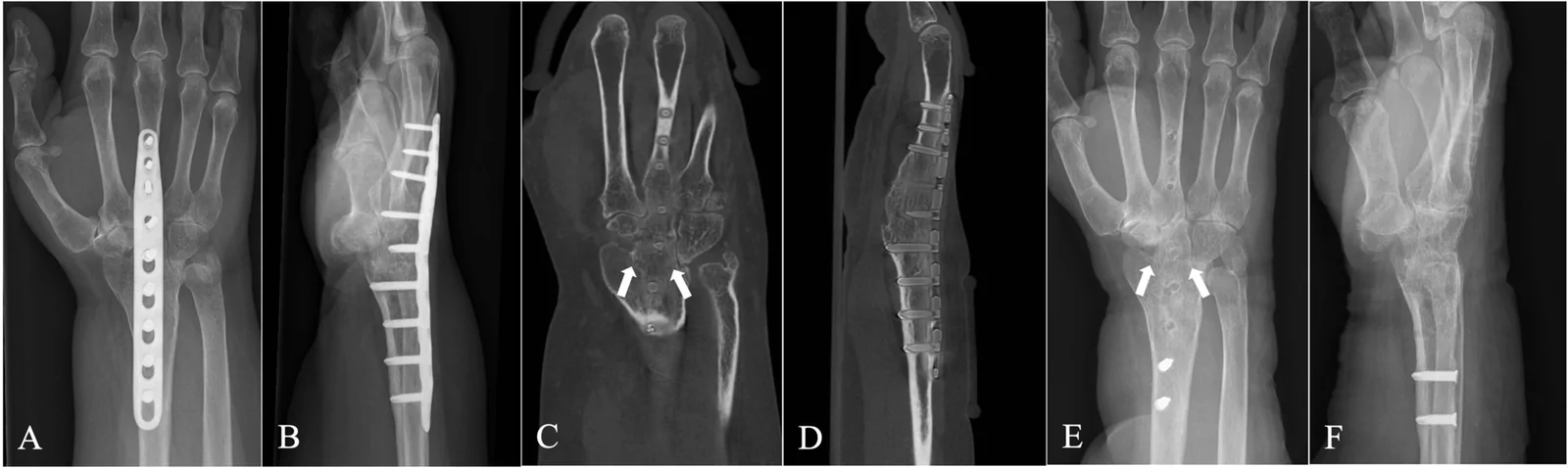
Shortening total wrist arthrodesis: standard radiographic and postoperative CBCT imaging. **A**,** B** Standard posteroanterior (**A**) and lateral (**B**) radiographs showing initial fixation with the wrist in slight extension (~ 10°), appropriate alignment, and no osseous displacement. **C**,** D** Coronal (**C)** and sagittal (**D**) CBCT reconstructions confirming solid fusion at the radiocapitate joint (arrows), with no abnormal motion or periprosthetic radiolucency. **E**,** F** Standard posteroanterior (**E**) and lateral (**F**) radiographs after hardware removal, demonstrating definitive fusion with maintained functional alignment and no radiographic complications

Other surgical options include shortening total wrist arthrodesis and implant-based procedures (Figs. 17,19). Shortening arthrodesis is typically indicated following failed PRC and involves creating a cavity within the distal radius to seat the capitate, followed by fixation in slight wrist extension [28].

Total wrist arthroplasty has historically been reserved for elderly patients with low physical demands and adequate bone stock [85]. Recent advances in implant design and long-term outcome data have expanded the indications to selected patients with post-traumatic and degenerative wrist osteoarthritis seeking pain relief while preserving wrist mobility, particularly extension, which facilitates digital flexion and functional grip [87–89]. Long-term comparative data now demonstrate that total wrist arthrodesis provides sustained improvement in pain, grip strength, and patient-reported functional outcomes at 10 years, with preservation of preoperative range of motion, contrasting with the predictable but motion-sacrificing outcome of arthrodesis [87]. However, these functional advantages are counterbalanced by higher complication and reoperation rates, particularly in younger or high-demand patients, underscoring the importance of strict patient selection and shared decision-making. Radial and carpal components are most often cementless. Implant evolution is classically described in generations, including the first-generation Swanson silicone implant introduced in 1967, second-generation ball-and-socket or hemispheric designs, and third-generation systems such as the Biaxial, Trispherical, and Universal prostheses [90]. Fourth-generation implants aim to improve fixation and reduce instability through porous titanium surfaces promoting osseointegration, preservation of the distal radioulnar joint, and limited bone resection [82, 85, 90]. Currently available fourth-generation systems include Universal 2™, RE-MOTION™, Maestro™, Modular, and Motec® [82].

Standard radiography remains the cornerstone of postoperative imaging assessment. Radiographic evaluation should include analysis of component alignment, carpal height, and periprosthetic radiolucent lines in the neutral position, as well as identification of associated distal ulnar resections commonly performed for distal radioulnar joint osteoarthritis [90]. At 10-year follow-up, cumulative implant survival reaches approximately 90% when revision is used as the primary endpoint, but decreases substantially when radiographic loosening is included, with reported survival rates around 75% [87]. Importantly, radiographic loosening does not consistently correlate with pain, functional decline, or deterioration in patient-reported outcome measures, supporting a conservative imaging surveillance strategy rather than systematic revision in asymptomatic patients [87].

**Fig. 20:**
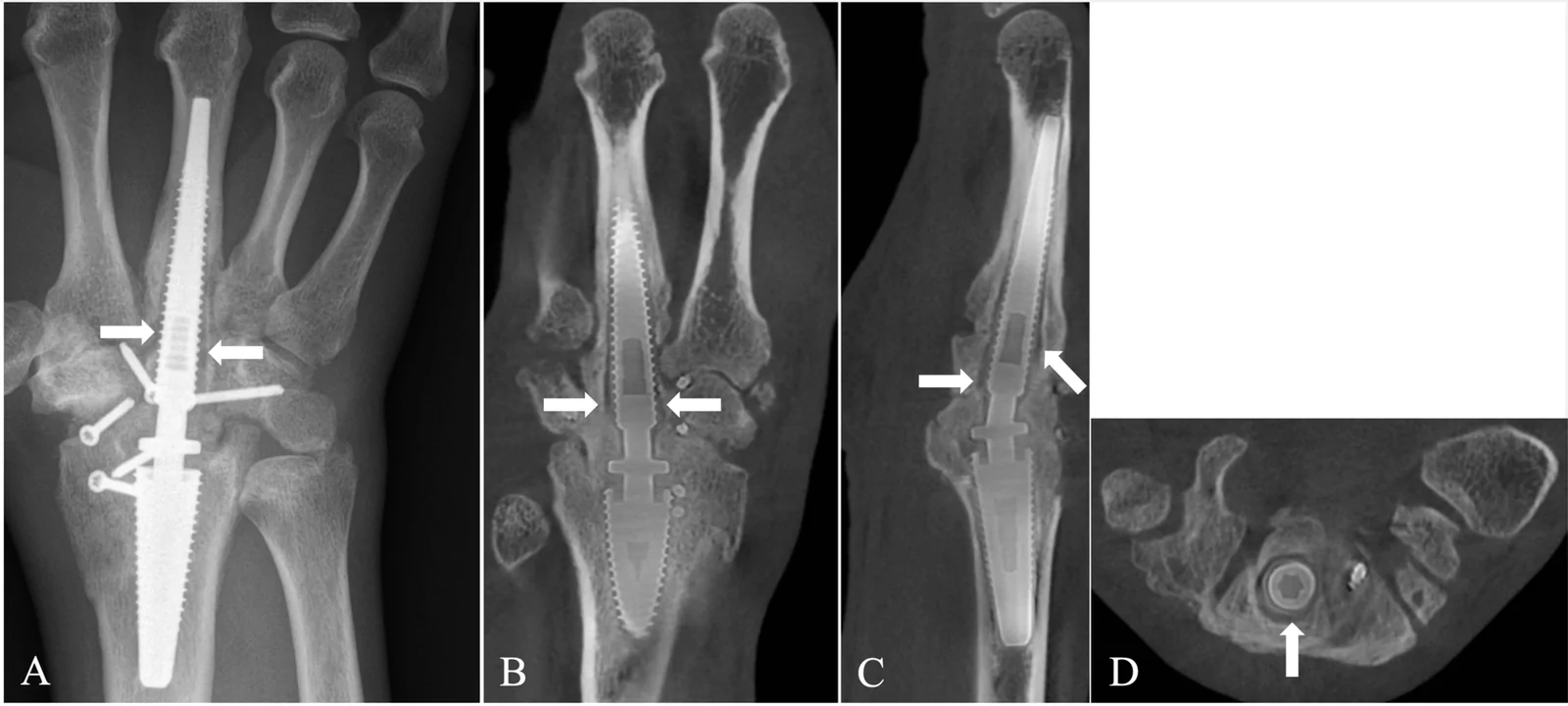
Loosening of a total wrist prosthesis. Posteroanterior radiograph (**A**) and coronal (**B**), sagittal (**C**), and axial (**D**) CBCT reconstructions. Radiolucent lines at the bone–prosthesis interface (arrows) indicate prosthetic loosening

Overall complication rates range from 6 to 47% [90, 91]. Early complications include intraoperative fractures, tendon injury, and wound-healing disorders, whereas late complications encompass periprosthetic fractures, loosening, migration, collapse, infection, polyethylene wear, metallosis, instability, and dislocation. Distal radioulnar joint-related complications include impingement, instability, and secondary osteoarthritis. Instability and component loosening represent the most frequent failure mechanisms (Fig. 20) [76]. Long-term studies confirm that periprosthetic radiolucency greater than 2 mm, although frequent, is often clinically silent, reinforcing the need for careful longitudinal correlation between imaging findings and clinical status rather than isolated radiographic decision-making [87, 92]. Revision total wrist arthrodesis or conversion from prior arthrodesis may be considered in specialized centers, providing meaningful pain relief and functional improvement; however, revision total wrist arthrodesis is consistently associated with higher reintervention rates and less predictable outcomes than primary arthroplasty, and current evidence increasingly favors conversion to radiocarpal arthrodesis after failed total wrist arthrodesis in many cases [87–89].

Radial hemiarthroplasty articulating with the capitate has been proposed as an alternative to total wrist arthroplasty to reduce the risk of carpal component loosening [65, 93]. Although minor complications such as contractures, superficial infection, and nerve compression are relatively common, major complication rates appear implant-dependent and may be substantial with certain systems [94].

Finally, massive pyrocarbon implants such as Amandys® have recently emerged as motion-preserving alternatives for stage 4 arthritic collapse, offering partial pain relief and preservation of limited wrist mobility in carefully selected patients, although long-term outcome data remain limited [95].

Table 4 presents the preferred interventions and post-operative radiological monitoring for SLAC/SNAC stage 4.

**Table 4:** SLAC / SNAC 4 [28, 47, 56, 76–95]

Stage	Primary Treatment	Alternatives/Options	Post-Treatment Imaging	Main Complications
SLAC/SNAC 4	Total wrist arthrodesis (radius, capitate, 3rd metacarpal ± additional carpal joints); adjunctive bone grafting if required; dorsal compression plate fixation; locked intramedullary wrist arthrodesis as an alternative	Shortening total wrist arthrodesis; total wrist arthroplasty (fourth-generation implants: Universal 2™, RE-MOTION™, Maestro™, Modular, Motec®); radial hemiarthroplasty; massive pyrocarbon implant (Amandys®)	Standard radiographs to assess fusion, alignment, wrist position (≈10° dorsiflexion), carpal height, hardware integrity, component positioning, and periprosthetic radiolucent lines; CT if nonunion or complications are suspected	Pseudarthrosis; infection; sensory nerve injury; carpal tunnel syndrome; hardware-related complications (migration, loosening, fracture); ulnocarpal impaction; distal radioulnar joint osteoarthritis; tendon adhesions; symptomatic dorsal plate prominence (bursitis, extensor tenosynovitis); complex regional pain syndrome; implant-specific complications; periprosthetic radiolucency
**Notes**	Reserved for patients with high functional demands or extensive pancarpal destruction; strict patient selection is essential; long-term follow-up recommended to monitor consolidation, implant stability, and adjacent joint degeneration			

## Conclusion

Post-traumatic periscaphoid osteoarthritis, represented by SLAC and SNAC stages, requires surgical management tailored to the extent of joint collapse. Postoperative evaluation relies on thorough knowledge of surgical techniques and implanted hardware, which is essential for accurate radiological interpretation. Various imaging modalities—including radiography, CT/CBCT, ultrasound, and MRI—play complementary roles in follow-up, enabling precise assessment of bone consolidation, implant positioning, and the early or late detection of complications. A targeted, multimodal imaging approach optimizes both clinical and radiological monitoring, guiding therapeutic decisions and enhancing functional outcomes.

## Data Availability

Not applicable.

## Abbreviations

CT: Computed tomography
DIC: Dorsal intercarpal ligament
DISI: Dorsiflexed intercalated segment instability
DRC: Dorsal radiocarpal ligament
dST: Dorsal scaphotriquetral ligament
LRL: Long radiolunate ligament
MRI: Magnetic resonance imaging
PRC: Proximal row carpectomy
SL: Scapholunate
SLAC: Scapholunate advanced collapse
SLIL: Scapholunate interosseous ligament
SNAC: Scaphoid nonunion advanced collapse
STT: Scaphotrapeziotrapezoid ligament
